# CdWRKY2 transcription factor modulates salt oversensitivity in bermudagrass [*Cynodon dactylon* (L.) Pers.]

**DOI:** 10.3389/fpls.2023.1164534

**Published:** 2023-07-17

**Authors:** An Shao, Xiao Xu, Erick Amombo, Wei Wang, Shugao Fan, Yanling Yin, Xiaoning Li, Guangyang Wang, Hongli Wang, Jinmin Fu

**Affiliations:** Coastal Salinity Tolerant Grass Engineering and Technology Research Center, Ludong University, Yantai, Shandong, China

**Keywords:** bermudagrass, WRKY, auxin, ABA, salt stress, lateral roots

## Abstract

Common bermudagrass [*Cynodon dactylon* (L.) Pers.] has higher utilization potential on saline soil due to its high yield potential and excellent stress tolerance. However, key functional genes have not been well studied partly due to its hard transformation. Here, bermudagrass “Wrangler” successfully overexpressing *CdWRKY2* exhibited significantly enhanced salt and ABA sensitivity with severe inhibition of shoot and root growth compared to the transgenic negative line. The reduced auxin accumulation and higher ABA sensitivity of the lateral roots (LR) under salt stress were observed in *CdWRKY2* overexpression *Arabidopsis* lines. IAA application could rescue or partially rescue the salt hypersensitivity of root growth inhibition in *CdWRKY2*-overexpressing *Arabidopsis* and bermudagrass, respectively. Subsequent experiments in *Arabidopsis* indicated that CdWRKY2 could directly bind to the promoter region of *AtWRKY46* and downregulated its expression to further upregulate the expression of ABA and auxin pathway-related genes. Moreover, *CdWRKY2* overexpression in *mapk3* background *Arabidopsis* could partly rescue the salt-inhibited LR growth caused by *CdWRKY2* overexpression*.* These results indicated that CdWRKY2 could negatively regulate LR growth under salt stress *via* the regulation of ABA signaling and auxin homeostasis, which partly rely on AtMAPK3 function. CdWRKY2 and its homologue genes could also be useful targets for genetic engineering of salinity-tolerance plants.

## 1 Introduction

Breeding and selection of salt-tolerant plant germplasm is a requisite for developing and utilizing saline-alkali land. Common bermudagrass [*Cynodon dactylon* (L.) Pers.] is an extensively used turfgrass and forage species due to its high stress tolerance level and high yield potential (Ackerson and Youngner, 1962). Despite having salt tolerance ability, the genetic mechanism underlying the salt response in bermudagrass is poorly understood, greatly limiting its utilization in saline soil. Therefore, it is necessary to isolate potential salt-responsive genes of bermudagrass and intensively investigate their salt-responsive function to provide useful gene resources for conducting high salt-tolerant and agronomical desirable bermudagrass varieties (lines).

WRKY transcription factors (TFs), named after their conserved amino acid sequences WRKYGQK, are vital for plant environmental stress response primarily by binding to the W-box elements at the promoter regions of downstream genes and then activating or inhibiting their transcription to active the following stress response cascade (Jiang et al., 2017). In model plants such as *Arabidopsis*, hormones such as abscisic acid (ABA) and auxin have previously been reported to play critical roles in environmental abiotic stress response. Also, a series of TFs could function in stress response *via* an ABA-dependent manner and regulation of auxin homeostasis including WRKY TFs (Zhou et al., 2008; Jiang and Deyholos, 2009; Lavenus et al., 2013; Cai et al., 2017; Jiang et al., 2017). For instance, AtWRK18, AtWRKY60, and AtWRKY40 interact physically and functionally to bind to the promoters of ABA response genes *ABI4* and *ABI5* (ABA-Insensitive 4 and 5) to suppress their expression (Liu et al., 2012). The *wrky18* and *wrky60* mutants exhibited a reduced ABA and salt sensitivity (Chen et al., 2010). The overexpression of *AtWRKY33* in *Arabidopsis* improves the salt-resistant ability of plants through the SOS (salt overly sensitive) pathway and increases plants’ sensitivity to ABA. The AtWRKY33 can also directly bind to the promoters of key enzymes encoding genes involved in ethylene biosynthesis (ACS2 and ACS6) to promote their expression, and then regulate ethylene biosynthesis induced by glutathione under salt conditions (Datta et al., 2015). Furthermore, other WRKY TFs have been reported to respond to salt stress *via* an ABA-independent manner. For example, under high salt stress in *Arabidopsis*, an AtWRKY8 directly binds to the W-box of the stress-responsive marker gene *RD29A* and promotes its expression to offset the Na^+^ and K^+^ homeostasis. The VQ9 can interact with AtWRKY8 and adversely affect its transcription activation function. However, compared to the wild type, salt stress significantly changes the expression of ABA response-related genes in *wrky8* and *vq9* mutants (Hu et al., 2013), suggesting that the effect of WRKY8 on salt tolerance does not depend on the ABA pathway. A recent study showed that a WRKY TF, AtWRKY46, binds to the promoter of genes involved in auxin conjugation and inhibits their expression to increase the active auxin accumulation in roots and maintain roots growth under salt stress (Korasick et al., 2013; Ding et al., 2015). Moreover, ABA and auxin pathways may have a combined effect in a wide range when modulating plant growth response to environmental stress (Smet et al., 2006). For example, ABA might regulate lateral root (LR) growth by affecting auxin transport (Brady et al., 2003; Deak and Malamy, 2005). Furthermore, ABI4 limits the expression of the auxin-efflux carrier protein PIN1 to inhibit the transporting of auxin to LR and mediates ABA-dependent inhibition of LR formation (Shkolnik-Inbar and Bar-Zvi, 2010). AtWRKY46 has also been reported to bind directly to the *ABI4* promoter to reduce its expression in *AtWRKY46* overexpression *Arabidopsis* plants grown in osmotic/salt stress conditions, suggesting that AtWRKY46 mediates the regulation of LR growth under osmotic/salt stress *via* effects on ABA signaling and modulation of auxin distribution in the roots (Ding et al., 2015).

MAPK (mitogen-activated protein kinase) cascade plays a vital role in plant response to stress including salt (Asai et al., 2002; Kim and Zhang, 2004; Andreasson et al., 2005). Generally, the signal molecules upstream activate MAPK cascade and lead to the phosphorylation of MAPKs to further activate them. The activated MAPKs often induce their translocation from the cytoplasm into the nucleus, where they can phosphorylate and activate sets of stress-resistant TFs (Shen et al., 2012). Some WRKY TFs have been reported to act downstream of MAPK cascades in plants. The binding ability of WRKY to the W-box motif of downstream genes can be enhanced or activated to further function in various physiological responses (Kim and Zhang, 2004). Among those MAPKs, MAPK3 and MAPK6 are the two main kinases that mediate downstream signaling in *Arabidopsis*. A previous study revealed that 26 out of 72 WRKY members of *Arabidopsis* interacted with MAPK3 (Sharma et al., 2013). For instance, in *Arabidopsis*, AtWRKY22 and AtWRKY29 function downstream of the MEKK1-MKK4/5-MPK3/6 cascade in response to elicitation by flagellin (Asai et al., 2002). AtWRKY33 can be directly phosphorylated by AtMPK3/AtMPK6 to regulate the expression level of genes that participated in camalexin biosynthesis in response to *Botrytis cinerea* infection. The mutation of the phosphorylation sites of AtWRKY33 compromises its camalexin induction ability (Mao et al., 2011).

Considering the crucial role of WRKY TFs in salt response, efforts have been made to investigate the potential function of WRKY genes in crops. For example, transgenic *Arabidopsis* overexpressing soybean *GmWRKY54* or wheat *TaWRKY2* in *Arabidopsis* induced the expression level of stress-responsive genes and thus elevated their salt tolerance ability (Zhou et al., 2008; Tao et al., 2011; Niu et al., 2012). Also, overexpressing cotton *GhWRKY34* in *Arabidopsis* could improve the salt tolerance of transgenic plants by protecting ionic balance and maintaining the stability of the intracellular environment (Zhou et al., 2015). Overexpressing *NbWRKY79* in tomatoes could reduce the accumulation of reactive oxygen and malonaldehyde, enhance the activity of antioxidant enzyme, and ultimately improve the salt tolerance (Nam et al., 2017). Recently, some crops expressing *WRKY* genes under salt stress followed the ABA signaling pathways. For instance, rice overexpressing *OsWRKY45-1* exhibited an induced expression of ABA signal-related and stress-responsive-related genes, which enhanced salt tolerance in the transgenic lines (Tao et al., 2011). Also, *Nicotiana* overexpressing a cotton *GhWRKY17* exhibited an enhanced salt and drought sensitivity by reducing ABA level and downregulating the expression of a series of ABA-inducible genes (Yan et al., 2014). Transgenic *Arabidopsis* overexpressing a GhWRKY6-like TF showed an improved salt tolerance by activating the ABA signaling pathway and decreasing the content of reactive oxygen species (Ullah et al., 2018). Overexpressing the maize *ZmWRKY17* in *Arabidopsis* enhanced salt sensitivity and decreased ABA sensitivity of transgenic plants by regulating ABA- and stress-responsive genes (Cai et al., 2017). Soybean GmWRKY16 promoted both salt and drought tolerance of transgenic *Arabidopsis* plants through an ABA-mediated pathway (Ma et al., 2019).

Generally, in tandem with their upstream regulators and interacting proteins, the WRKY TFs can modulate plants’ salt response by activating or repressing the expression of downstream salt-responsive genes, regulating hormone-related pathways, or inducing physiological responses (Jiang et al., 2017). Partly due to the lack of full genomic sequence information and its hard transformation, the functional study of salt-responsive *WRKY* genes in bermudagrass for potential cultivation in saline soil has been limited. Therefore, in this study, based on the efficient and stable genetic transformation system established in our laboratory, the salt-responsive function and possible molecular mechanism of a salt-induced WRKY TF encoding bermudagrass *CdWRKY2* gene was intensively investigated. Transgenic bermudagrass and *Arabidopsis* plants overexpressing *CdWRKY2* showed higher salt-induced inhibition of LR growth, decreased salt tolerance, and increased ABA sensitivity compared to the negative control plants. Our results demonstrated that CdWRKY2 and its homologues can be useful targets for genetic engineering of salinity-tolerance varieties to achieve salt-tolerant germplasm breeding.

## 2 Materials and methods

### 2.1 Plant materials

The bermudagrass variety “Wrangler” was used to isolate gene sequence and check gene expression pattern and was used for transgenic donor. The wild type Col-0 and *mapk3* mutant were used to create the overexpression *Arabidopsis* lines. The *DR5::GUS* lines were used to cross with the *CdWRKY2* overexpression line to generate *DR5::GUS* lines under the *CdWRKY2* overexpression background.

### 2.2 Gene identification and phylogenetic analysis

The sequence of the *CdWRKY2* coding sequence was identified and isolated from our transcriptome data by using the primers supplied in **Table S1**. Using ClustalX 2.0, CdWRKY2 and WRKY proteins from other species were aligned (Thompson et al., 2002). Using the neighbor-joining method, phylogenetic trees were conducted and were displayed using the MEGA 5.0 software (Tamura et al., 2011).

### 2.3 Vector construction and transformation of plants

The *CdWRKY2* coding region was inserted into an entry vector PGWC to construct *PGWC-CdWRKY2* using the homologous recombination method. Then, the entry clone *PGWC-CdWRKY2* was mixed with a Gateway^®^ vector *pUbi-6B* for LR reaction to generate expression vector *pUbi::CdWRKY2*. The construct was then transformed into immature embryos of the bermudagrass variety “Wrangler”. To generate the *p35S::CdWRKY2* construct, the *CdWRKY2* coding sequence was cloned into the *pRI101-AN* vector. An *Agrobacterium* strain GV3101 containing the constructed vector was then transformed into *Arabidopsis via* a floral dip method. The homozygous T3 seedlings of independent transgenic lines were used for further experiments. The primers used for vector construction for transformation are listed in **Table S1**.

### 2.4 Hydroponic culture of bermudagrass

The uniform stolons of the bermudagrass wild-type “Wrangler” and overexpression bermudagrass lines were planted in solid medium (sand with nutrient solution/1/2 Hogland) for a month. For the RT-qPCR analysis of salt response, the seedlings were washed clean and transferred into CK (1/2 Hogland with 0 mM NaCl) and salt stress (1/2 Hogland with 200 mM NaCl) for 0, 6, or 12 h in the shoots and roots, and the seedlings were transferred into CK (0 mM NaCl) and salt stress (200 mM NaCl) for 0, 6, or 12 h, respectively. For the expression analysis of ABA response, the seedlings were then transferred into 1/2 Hogland with 0 μM ABA or 100 μM ABA added for 0, 3, 6, 12, or 24 h, respectively. For phenotype characterization, after being planted in a single branch of the wild type, overexpression lines were planted in solid medium for approximately 7–10 days. The roots of each plant were washed clean and then supplied with 0 mM NaCl (CK) or 200 mM NaCl (Salt) for approximately 20 days in hydroponic culture. The hydroponic culture was processed in a growth chamber. The growth conditions were set as follows: 65% relative humidity, 400 µmol m^−2^ s^−1^ photons and 22/18°C (16-h day/8-h night). The roots and shoots from three biological replicate plants under control and salt conditions were harvested separately for the analysis of physiological parameters and root morphology. Before treatment and after treatment, the biomass of individual samples was measured and the total PR length, total root length, and number were determined using Win-RHIZO software (Regent Instruments Canada Inc., Ottawa, ON, Canada) to further calculate the relative increment of these parameters. In a separate experiment, the seedlings were transferred to 1/2 Hogland containing 200 mM NaCl (Salt), 200 mM NaCl with 10 nM IAA (Salt+IAA), or 100 μM ABA and grown for approximately 2 weeks to determine the relative increase of root growth parameters, respectively.

### 2.5 Pot experiment of bermudagrass

The wild-type “Wrangler” and transgenic bermudagrass lines were cut to the same height. The experiment consisted of control and salt treatment conditions, each of which had three biological replications. For salt treatment of bermudagrass, the plants were treated with 200 mM NaCl for 4 weeks. For the control condition, the plants were supplied with water without NaCl. The plant height and biomass of every individual plant from the three biological replicates was detected and the leaves and roots were harvested separately to determine the physiological parameters. The electrolyte leakage (EL), malondialdehyde (MDA), and the activities of antioxidant enzymes were measured based on the previous description (Shao et al., 2020).

### 2.6 Plant growth conditions of *Arabidopsis*

CK (0 mM NaCl or ABA) represents normal growth conditions for *Arabidopsis*, and Salt (100 mM NaCl) or ABA (10 μM) represents stress treatment. Briefly, the stratified *Arabidopsis* seeds were cultured on nutritious medium (one-half-strength Murashige and Skoog) containing 1.0% agar for 4 days. The seedlings were then transferred to CK, Salt, and ABA medium and were cultured vertically for 7–10 days in a growth chamber, which was set at 22°C with a 16-h/8-h (light/dark) cycle. The numbers of visible lateral roots were counted manually. After salt treatment, the shoots of Col-0 and transgenic *Arabidopsis* lines were collected to measure their shoots’ fresh weight. The root samples of Col-0 and transgenic lines were collected for gene expression analysis. In a separate experiment, the seedlings were transferred to 1/2 MS containing 100 mM NaCl (Salt) or 100 mM NaCl with 0.1 μM IAA (Salt+IAA) for shoot fresh weight and LR number measurement, respectively.

### 2.7 Yeast transformation

For the trans-activation assay, the coding region of *CdWRKY2* was amplified and cloned into the *pGBKT7* vector (Clontech) to generate a *pGBKT7-CdWRKY2* construct and was then transformed into yeast strain AH109 competent cells. The empty *pGBKT7* vector was transformed individually as a negative control. The transformed yeast cells were cultured on SD/-Trp/-His/-Ade to check their growth status. For DNA binding activity, three W-box (CGTTGACC) and three mutated W-box (mW-box) (CGTAGACG) were constructed into the *pAbAi* vector, respectively, and were grown on growth medium containing 800 ng/ml AbAi. For yeast two-hybrid, the *pGBKT7-CdWRKY2* and *pGADT7-MAPK3* (or *pGADT7-AtWRKY46*) vectors were co-transformed into Y2H gold strain competent cells. The *pGBKT7-53* and *pGADT7-T* vector pair was co-transformed as a positive interaction control. The transformed yeast cells were grown on SD/-Trp/-Leu and SD/-Trp/-Leu/-Ade/-His plates, respectively. For yeast one-hybrid, the W-box-containing region of *ABI4*, *UGT84B2*, and *AtWRKY46* promoter sequence was cloned into *pAbAi* yeast vector to construct *pABI4-AbAi*, *pUGT84B2-AbAi*, and *pAtWRKY2-AbAi* vectors. The *pGADT7-CdWRKY2/pABI4-AbAi* (or *pUGT84B2-AbAi* or *pAtWRKY46*) vector pair was co-transformed into Y1H yeast strains, respectively. Furthermore, the *pGADT7-AtWRKY2* and *pAtWRKY46* vector pair was co-transformed into Y1H yeast strains. All the yeast cells were streaked on SD/-Leu and SD/-Leu+AbAi plates (200 ng/ml), respectively, and were placed at 28°C under dark conditions for 3–5 days to check their growth state.

### 2.8 GUS staining and GUS quantitative assay

DR5::GUS staining in *Arabidopsis* samples was conducted as previously described (Ma et al., 2014). Samples were stained in solution containing 50 mM NaPO_4_, 0.4 mM K_3_Fe(CN)_6_, 0.4 mM K_4_Fe(CN)_6_, 1 mM X-gluc, and 0.1% Triton X-100 incubated at 37°C for 8 h in the dark. After GUS staining, the stained samples were washed with 70% ethanol three times and then microscopically imaged. GUS activities were measured as previously described (Liu et al., 2015). Briefly, **s**eedlings of each sample were collected in Eppendorf tubes (100 mg per measurement) and homogenized with steel beads in buffer (50 mM potassium phosphate buffer, pH 7, 1 mM EDTA, 0.1% SDS, and 0.1% Triton X-100). The supernatant was used for measurement after centrifuging at 12,000 rpm for 15 min at 4°C and the protein concentrations were quantified using a Micro BCA Protein Assay Kit (Vigorous). Enzyme activity was calibrated by the concentration of 4-methylumbelliferone (Sigma-Aldrich). The fluorescence of the samples was measured on 96-well plates on a Fluoroskan Ascent FL fluorometer (excitation wavelength of 365 nm and emission wavelength of 455 nm) after incubating with 4-methylumbelliferyl-β-D-glucuronide hydrate (Sigma-Aldrich). Measurements were read when stopped by adding stop buffer (0.2 M Na_2_CO_3_) at 0, 15, 45, and 60 min, and the standard curve was fitted.

### 2.9 Gene expression analysis

Total RNA of different tissues was extracted (RNAeasy kit, QIAGEN) and the commensurable RNA of each sample was reverse-transcribed to first-strand cDNA and were diluted to the same multiples for the template for gene expression analysis (TaqMan reverse transcription kit, Applied Biosystems). The qPCR system containing 10 μl of SYBR Green mix (Roche, Mannheim, Germany), 2 μl of template, and 0.2 μM primers was conducted using the ABI real-time PCR system (Applied Biosystems, Foster City, CA). Three technical replicates of each reaction were performed. Transcript levels of each sample were determined and normalized with respect to the internal control gene using the ΔΔCt method (Czechowski et al., 2005; Schmittgen and Livak, 2008; Chen et al., 2015). The specific primers used for RT-qPCR are shown in **Table S2**.

### 2.10 Statistical analysis of data

SPSS22 software for Windows (SPSS Inc., Chicago, Illinois, US) was used for one- or two-way analysis of variance (ANOVA tests, Tukey’s post‐hoc test). The individual *p* level of comparison is shown in **Tables S3**, **S4**, and **S5**.

## 3 Results

### 3.1 Identification, expression, and DNA binding activity analysis of CdWRKY2

**Figure 1:**
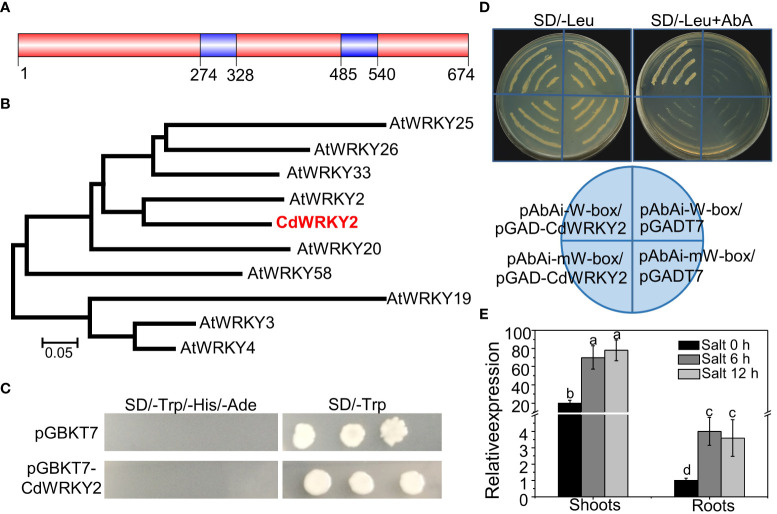
Sequence analysis, transcription activation activity, DNA binding activity, and gene expression of CdWRKY2. **(A)** The protein structure of CdWRKY2 with two WRKY domains. **(B)** Phylogenetic tree constructed with CdWRKY2 and other WRKY proteins in *Arabidopsis* by the MEGA 5.0 program with neighbor-joining method. **(C)** Transcriptional activation analysis of CdWRKY2 in yeast cells. The negative control vector was *pGBKT7*. **(D)** W-box and mutated W-box (mW-box) was constructed into *pAbAi* vector, respectively, and were used to check DNA binding activity of CdWRKY2 using the yeast one-hybrid method. RT-qPCR analysis of *CdWRKY2* expression in the shoots and roots under salt condition (200 mM NaCl for 0, 6, and 12 h) **(E)**. The internal control gene used for salt treatment was *CdPP2A*. Data are represented as means ± SD of three independent replicates, and different letters indicate significant differences at *p* < 0.05 by two -way analysis of variance (ANOVA tests) with Tukey’s post‐hoc test.

Here, a *CdWRKY2* gene, containing a complete open reading frame (ORF) of 2,025 bp, was cloned from bermudagrass. The CdWRKY2 contained two WRKYGQK motifs (**Figure 1A**) and a C_2_HC-type zinc finger and belonged to the group I WRKY subfamily (**Figure S1**). Homology search and phylogenetic analysis of the WRKY-related proteins against the GenBank database showed that CdWRKY2 was homologous to AtWRKY2 TF of *Arabidopsis* (**Figure 1B**). A transcriptional activity assay using yeast one-hybrid method indicated that CdWRKY2 possesses no transcriptional activation activity in the yeast. This was deduced from the observation that only the positive control yeast cells could grow on the SD/-Trp/-His/-Ade, which was converse to the negative control and the cells transformed with *pGBKT7-CdWRKY2* (**Figure 1C**). However, CdWRKY2 had a putative role as a WRKY TF binding to the W-box of target genes because the *pAbAi-W-box*/*pGADT7-CdWRKY2* co-transformation yeast cells could grow on SD/-Leu (containing 800 ng/ml AbA) medium whereas the *pAbAi-mW-box*/*pGADT7-CdWRKY2* (mW represents mutated W-box) co-transformation yeast cells could not grow (**Figure 1D**). A quantitative real-time PCR (RT-qPCR) analysis indicated that the *CdWRKY2* was expressed in both the roots and shoots (**Figure 1E**). Also, the investigation of expression pattern in response to salt revealed a strong upregulation of *CdWRKY2* expression in the shoots and roots after NaCl exposure for 6 h or 12 h (**Figure 1E**).

### 3.2 Overexpressing *CdWRKY2* in bermudagrass increases plants’ salt sensitivity

**Figure 2:**
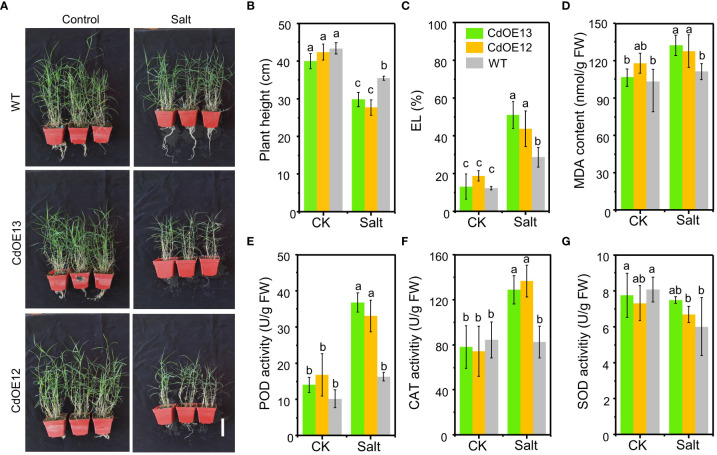
The phenotype of *CdWRKY2* overexpression transgenic bermudagrass plants. The same number of branches of the *CdWRKY2* overexpression lines (CdOE13 and CdOE12) and the transgenic negative control line (WT) were planted in soil for about 1 month. The plants were cut to the same height and supplied with 0 mM NaCl (CK) or 200 mM NaCl (Salt) and then treated for 4 weeks. **(A)** Images of the overexpression lines and control line grown under CK and salt conditions. Bar = 10 cm. **(B)** Plant height after treatments. **(C)** EL. **(D)** MDA content. **(E)** POD activity. **(F)** CAT activity. **(G)** SOD activity. Data are the means ± SD of three independent biological replicates containing at least 10 plants. Different letters represent statistically significant differences at *p* < 0.05. Two-way ANOVA test was used and the data were further compared by Tukey’s post‐hoc multiple range test. Different letters on histograms indicate that means were statistically different at the *p* < 0.05 level.

By transforming the bermudagrass cultivar “Wrangler” with *pUbi::CdWRKY2*, eight overexpression lines were successfully obtained (**Figure S2**) and two lines (CdOE13 and CdOE12) with higher *CdWRKY2* expression levels were selected for subsequent investigation under control and salt conditions (**Figures 2A**; **S2**). In the pot experiment, the plant height of the two overexpression lines (CdOE13 and CdOE12) and the negative control plants (WT) showed no significant difference. However, under salt conditions, overexpression of *CdWRKY2* in bermudagrass significantly decreased plant height compared to the negative control plants (**Figure 2B**). The salt tolerance-related physiological indicators were further measured. We observed that the EL (**Figure 2C**) and MDA content (**Figure 2D**) of *CdWRKY2* overexpression lines were significantly higher than those of the negative control plants under salt conditions. Moreover, we determined the degree of lipid peroxidation and the activities of antioxidant enzymes of the leaves. Under salt stress, the activities of peroxidase (POD) (**Figure 2E**) and catalase (CAT) (**Figure 2F**) were greater than those of the negative control plants while the superoxide dismutase (SOD) activities remained statistically unchanged (**Figure 2G**). The phenotypic and physiological indicators of plants observed and detected under salt stress suggested that overexpression of *CdWRKY2* could increase the sensitivity of bermudagrass to salt stress.

### 3.3 *CdWRKY2* overexpression inhibits the salt-induced root growth of bermudagrass

**Figure 3:**
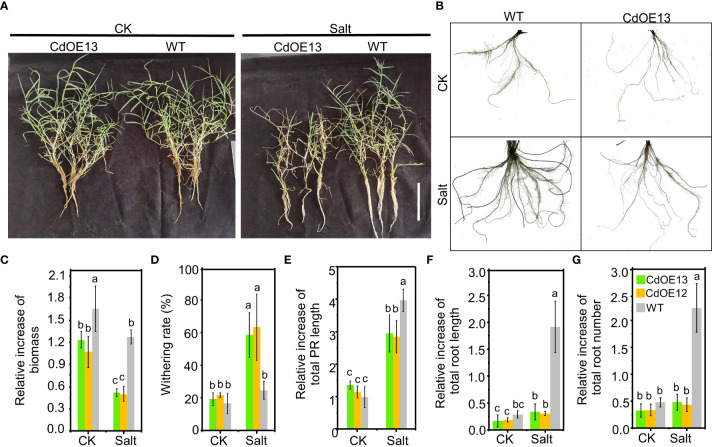
The phenotype of *CdWRKY2* overexpression transgenic bermudagrass grown in hydroponic culture. A single branch of the *CdWRKY2* overexpression lines (CdOE13 and CdOE12) and the transgenic negative control line (WT) was planted in soil for approximately 7 days. The roots of each plant were washed clean and then supplied with 0 mM NaCl (CK) or 200 mM NaCl (Salt) for 20 days in hydroponic culture. **(A)** Images of the overexpression lines and control line grown under CK and salt conditions. Bar = 10 cm. **(B)** Images of roots of plants under CK and salt conditions. **(C)** Relative increment of biomass before and after treatment. **(D)** Withering rate. **(E)** Relative increase of total PR length before and after treatment. **(F)** Relative increase of total roots length before and after treatment. **(G)** Relative increase of total roots number before and after treatment. Data are the means ± SD of three biological replicates. Two-way ANOVA test was used and the data were further compared by Tukey’s post‐hoc test. Significant differences at *p* < 0.05 were shown by different letters above the columns.

In the soil experiment, we noticed that salt stress notably induced the root growth of negative control plants while there was no obvious induction of root growth of overexpression lines (**Figure 2A**). To further confirm the inhibition of root growth in *CdWRKY2* overexpression lines, we hydroponically investigated the root growth difference. The results showed that under salt conditions, both the CdOE13 and CdOE12 lines exhibited a lower increase of biomass (**Figure 3A**) and a higher withering rate (**Figure 3B**) than the negative control plants, which was consistent with the soil experiment. Concretely, the negative control plants showed significantly increased total root length (**Figure 3F**) and total root number (**Figure 3G**) when exposed to salt conditions compared to the control condition. However, total root length and number of *CdWRKY2* overexpression lines showed no significant salt-induced increase under salt stress compared to the control condition (**Figures 3F, G**). In addition, under salt stress, the *CdWRKY2* overexpression-induced inhibition degree on the primary roots (PR) was relatively lower than that of the LR, as the salt-induced relative increment of total PR length (**Figure 3E**) and total root length (**Figure 3F**) of the overexpression lines was approximately 70% and 10% that of the wild type, respectively. This observation suggested that the salt-induced total root length increment might stem from the contribution of LR increase and the inhibition effect of overexpressing *CdWRKY2* on root growth under salt stress might be mainly derived from LR inhibition.

### 3.4 Overexpression of *CdWRKY2* inhibits LR growth and increases salt sensitivity in *Arabidopsis*

**Figure 4:**
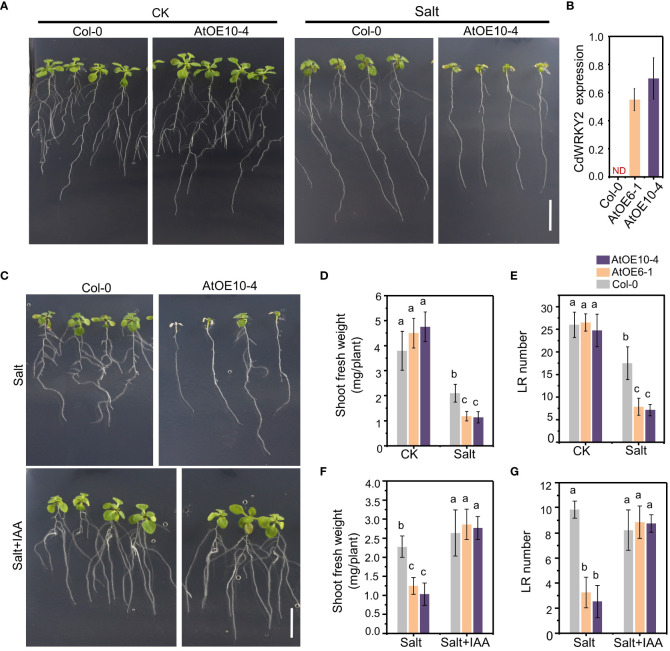
The phenotype of the *CdWRKY2* transgenic *Arabidopsis* lines. The seeds of wild type (Col-0) and *CdWRKY2* overexpression lines (AtOE10-4 and AtOE6-1) were germinated and grown for 4 days on 1/2 MS. The seedlings were transferred to 1/2 MS containing 0 mM NaCl (CK), 100 mM NaCl (Salt), or 100 mM NaCl with 0.1 μM IAA (Salt+IAA), respectively. The morphological parameters were measured after being grown vertically for 7 days. **(A)** Images of overexpression line (AtOE10-4) and wild type (Col-0) grown 7 days on CK and salt conditions. Bar = 1 cm. **(B)** Relative expression of *CdWRKY2* in AtOE10-4 and AtOE6-1. **(C)** Images of AtOE10-4 and Col-0 grown 7 days on 1/2 MS containing salt and salt plus 0.1 μM IAA, respectively. Bar = 1 cm. **(D)** Shoot fresh weight of overexpression and wild-type plants grown under CK and salt conditions. **(E)** Visible LR number of overexpression and wild-type plants grown under CK and salt conditions. Shoot fresh weight **(F)** and visible LR number **(G)** of overexpression and wild-type plants grown under salt and salt plus IAA conditions. Data are represented as means ± SD of at least 15 seedlings from three independent treatments. Two-way ANOVA test was used and the data were further compared by Tukey’s post‐hoc test. Different letters displayed represent significant differences at *p* < 0.05 statistically.

To investigate the possible salt-induced mechanism underlying the root growth inhibition of *CdWRKY2* overexpression lines (AtOE10-4 and AtOE6-1), we studied the growth pattern of transgenic *Arabidopsis* lines overexpressing this gene (**Figures 4A, B**). Under salt conditions, the shoot and root growth of AtOE10-4 was obviously decreased than that of the wild type (Col-0) (**Figure 4A**). The results showed that both the AtOE10-4 and AtOE6-1 lines had lower shoot fresh weight (**Figure 4D**) compared to the wild type under salt treatment. Furthermore, salt treatment significantly decreased the number of visible LR in transgenic lines relative to the wild type (**Figure 4E**). Reports showed that the stress-induced LR inhibition can be realized by interfering with auxin-related response (Lavenus et al., 2013). Therefore, we added IAA (1 μM) into the growth media. Interestingly, we observed that IAA application could significantly rescue the salt hypersensitivity of salt-treated *CdWRKY2* transgenic *Arabidopsis* lines (**Figure 4C**), as the inhibition of LR growth (**Figure 4F**) and shoot growth (**Figure 4G**) showed no significant difference between overexpression lines and the wild type after IAA application. In bermudagrass, IAA application could partially rescue the salt hypersensitivity of root growth in *CdWRKY2*-overexpressing bermudagrass lines, as the salt-inhibited total root length and total root number were slightly decreased compared with the wild type (**Figures S3C, D**). From the results in bermudagrass together with *Arabidopsis*, CdWRKY2 was proved to participate in the inhibition of salt-induced LR growth in bermudagrass and in the salt-inhibited LR growth in *Arabidopsis*. Moreover, preliminary results indicated that CdWRKY2 might negatively affect LR development under salt stress and increase the salt sensitivity of plants *via* the regulation of auxin homeostasis.

### 3.5 IAA accumulation decreases in *CdWRKY2* overexpression *Arabidopsis*

**Figure 5:**
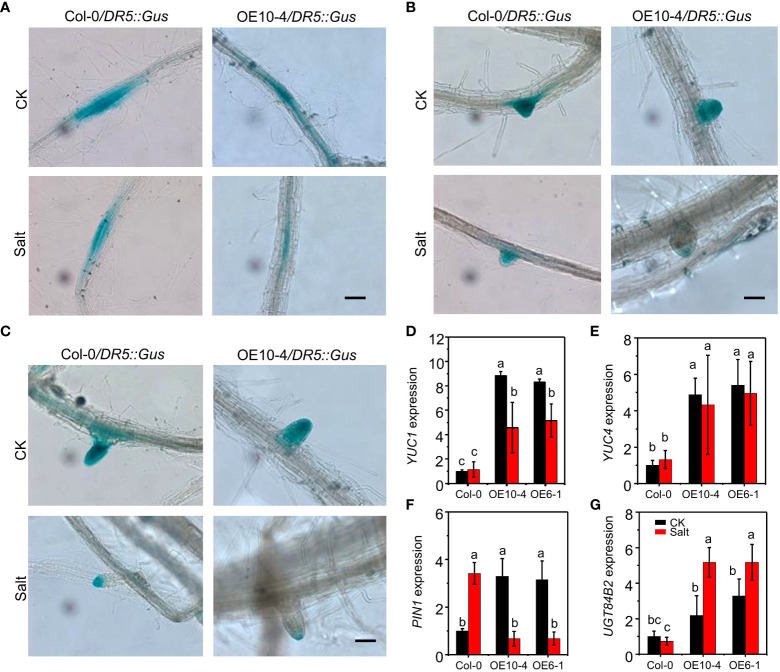
GUS staining and auxin pathway-related gene expression of Col-0 and the *CdWRKY2* overexpression background. The seeds of wild type (Col-0), *CdWRKY2*-overexpressing line (AtOE10-4), plants expressing *DR5::GUS* in Col-0 and AtOE10-4 background were germinated for 4 days. The seedlings were transferred to 1/2 MS that contained either 0 mM NaCl (CK) or 100 mM NaCl (Salt) for 7 days. GUS staining in LR primordia (LRP) **(A)**, emerged LR tip **(B)**, and visible LR **(C)**. Bar = 1000 μm. Gene expression of *YUC1* **(D)**, *YUC4* **(E)**, *PIN1* **(F)**, and *UGT84B2* **(G)** in Col-0 and AtOE10-4 roots under CK and salt conditions were determined by RT-qPCR using *UBQ10* as an internal control reference gene. Transcript levels of each sample were normalized to the expression of untreated wild-type plants. Three independent replicates were performed. Two-way ANOVA test was used and the data were further compared by Tukey’s post‐hoc test. Different letters represent significant differences at *p* < 0.05.

To determine the possible contribution of auxin distribution in salt-induced *CdWRKY2* inhibitory role to LR development, the expression pattern of *DR5::GUS* (Ulmasov et al., 1997) was investigated in both the wild type (Col-0) and *CdWRKY2* overexpression background (AtOE10-4) plants grown under both control and salt conditions. We observed that the overexpression line AtOE10-4 exhibited a weaker GUS staining in LR primordia (LRP) (**Figure 5A**), non-emerged LR tip (**Figure 5B**), and the visible LR tip (**Figure 5C**) compared with the wild type under salt stress. However, under normal conditions, GUS staining in LR showed no obvious difference between transgenic line and wild type (**Figure 5**). The relative GUS activity of roots expressing *DR5::GUS* in AtOE10-4 presented a significant lower salt-inhibited value compared to the wild type (**Figure S4**). The expression of auxin synthesis gene *YUC1* and *YUC4* was higher in overexpression lines under both control and salt conditions and significantly decreased by salt (**Figures 5D, E**). Furthermore, the expression of auxin transporter encoding gene *PIN1* in the transgenic lines was higher under normal conditions but decreased significantly under salt conditions (**Figure 5F**). *PIN3* was upregulated in the overexpression line under both control and salt conditions (**Figure S5A**). However, under both control and salt conditions, the expression of other auxin transport-related genes such as *PIN2*, *PIN7*, *PID* (**Figures S5B–D**), and other auxin synthesis-related genes *TAA1* and *TAR2* (**Figures S5E, F**) remained statistically unchanged in wild-type and transgenic lines. Moreover, salt significantly induced the expression of the auxin conjugation-related gene *UGT84B2* and had a higher level in *CdWRKY2* overexpression lines under both control and salt conditions (**Figure 5G**). Other auxin conjugation-related genes such as *IAGLU* and *GH3.1* showed no difference between wild-type and transgenic lines under both control and salt conditions (**Figures 5G, H**).

### 3.6 Root ABA sensitivity increases in *CdWRKY2* overexpression lines

**Figure 6:**
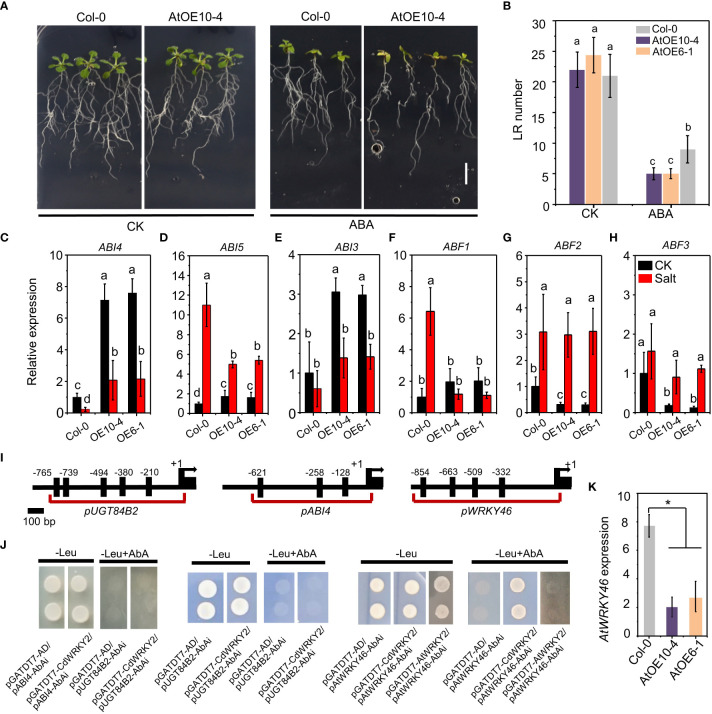
The ABA sensitive phenotype of the *CdWRKY2* overexpression *Arabidopsis* lines. The seeds of wild type (Col-0), *CdWRKY2* overexpression lines (AtOE10-4 and AtOE6-1) were germinated for 4 days on 1/2 MS and the seedlings were then transferred to 1/2 MS containing 0 mM ABA (CK) or 10 μM ABA (ABA), respectively. After being grown vertically for 10 days, morphological parameters were measured. **(A)** Images of overexpression line (AtOE10-4) and wild type (Col-0) grown 10 days on CK and ABA conditions. Bar = 1 cm. **(B)** Visible LR number of overexpression lines and wild-type plants grown under CK and ABA conditions. **(C–F)** Relative expression of ABA-responsive genes in the roots of Col-0 and overexpression lines under control and salt conditions of three independent replicates. *ABI4* **(C)**, *ABI5* **(D)**, *ABI3* €, *ABF1* **(F)**, *ABF2* **(G)**, *ABF3* **(H)**. *UBQ10* was used as an internal control reference gene. Transcript levels of each sample were normalized to the expression of untreated wild-type plants. Three independent replicates were performed. Two-way ANOVA test was used and the data were further compared by Tukey’s post‐hoc test. Different letters on histograms indicate that means were statistically different at the *p* < 0.05 level. **(I)** Illustration of the *ABI4*, *UGT84B2*, and *AtWRKY46* promoter regions showing the presence of consensus motif W-box. Shown are 2-kb upstream sequences of genes. **(J)** Yeast one-hybrid analysis of the binding of CdWRKY2 to the promoters. The promoter region of *ABI4*, *UGT84B2*, and *AtWRKY46* containing multiple W-box was constructed into pAbAi vector, respectively, and was used to check DNA binding activity of CdWRKY2 using yeast one-hybrid method. The promoter region of *AtWRKY46* was then used to check the DNA binding activity of AtWRKY2. **(K)** The relative expression level of *AtWRKY46* in *CdWRKY2* overexpression lines. Data are represented as means ± SD of three independent replicates, and * indicates significant difference between Col-0 and overexpression lines at *p* < 0.05 by one-way analysis of variance with Tukey’s post‐hoc test.

To investigate the role of ABA on salt-induced *CdWRKY2* overexpression, we compared the LR growth of wild type and *CdWRKY2* overexpression *Arabidopsis* lines on a medium containing 10 μM ABA. Results indicated that overexpression lines exhibited higher ABA sensitivity with less LR number (**Figures 6A, B**). In bermudagrass, the upregulated expression in the roots by ABA was observed especially after ABA application for 6 h and 12 h (**Figure S3B**). The ABA sensitivity *CdWRKY2* overexpression line also showed significant elevation compared to the wild type with decreased root length and root number (**Figures S3C, D**). In the *CdWRKY2* overexpression *Arabidopsis* lines, the expression of ABA-responsive genes such as *ABI4* (**Figure 6C**), *ABI5* (**Figure 6D**), *ABI3* (**Figure 6E**), and *ABF1* (**Figure 6F**) was upregulated compared to wild type under control conditions, which might contributed to the ABA hypersensitive phenotype of *CdWRKY2* overexpression lines. The *ABI4* (**Figure 6C**) gene was highly expressed in *CdWRKY2* overexpression lines compared to the wild type under both control and salt conditions. Under salt stress, the expression of *ABI5* (**Figure 6D**) and *ABF1* (**Figure 6F**) decreased in the *CdWRKY2* overexpression lines compared to that in the wild type, while under control conditions, the expression of *ABF2* (**Figure 6G**) and *ABF3* (**Figure 6H**) declined in the *CdWRKY2* overexpression lines compared to that in the wild type. In contrast, in transgenic lines, the expression of *ABI1* and *ABI2* showed higher salt inhibition degree compared to the wild type (**Figures S6A, B**). *ABI4* or *UGT84B2* was reported to act downstream of AtWRKY46 to play a critical role in LR growth under salt stress (Ding et al., 2015). Therefore, using the yeast one-hybrid system, we investigated the interaction of CdWRKY2 with their promoter regions, which contained several W-box (**Figure 6I**). However, *pGADT7-CdWRKY2* and *pABI4-AbAi* (or *pUGT84B2-AbAi*) co-transformed yeast cells could not grow on -Leu plates containing 200 ng/ml AbAi, the same as control vector pairs (*pGADT7-AD/pABI4-AbAi* or *pGADT7-AD/pUGT84B2-AbAi*), suggesting that there was no interaction *in vitro* (**Figure 6J**). However, in *CdWRKY2* overexpression lines, the *AtWRKY46* expression level was significantly inhibited compared to wild type (**Figure 6K**). Subsequently, yeast two-hybrid and yeast one-hybrid experiments showed that CdWRKY2 had no protein interaction with AtWRKY46 (**Figure S7**) but could bind to the promoter region of *AtWRKY46* (**Figure 6J**). Moreover, in the yeast one-hybrid assay, no interaction was observed between AtWRKY2 protein (the orthologs of CdWRKY2) and the promoter of *AtWRKY46*, suggesting that AtWRKY2 and CdWRKY2 might not be direct counterparts during salt-induced root inhibition (**Figure 6J**).

### 3.7 *CdWRKY2* functions in elevating salt sensitivity in *Arabidopsis* partially *via* AtMAPK3

**Figure 7:**
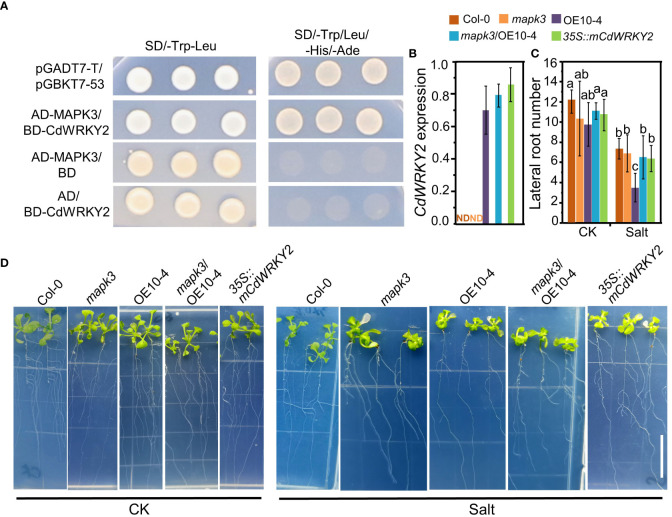
CdWRKY2 functions in LR growth inhibition under salt stress in *Arabidopsis via* the function of MAPK3. **(A)** Interactions between AtMAPK3 and CdWRKY2 in the yeast cell. Transformants transferred with different construct pair growth on SD/-Trp-Leu and SD/-Trp-Leu-His-Ade plates. *pGADT7-T*/*pGBKT7-53* was used as a positive control vector pair. **(B)** The seeds of Col-0, *mapk3* mutant, AtOE10-4 (*CdWRKY2* overexpression line), *mapk3*/AtOE10-4 (*CdWRKY2* overexpression in *mapk3* mutant background), and *35S::mCdWRKY2* (mutated *CdWRKY2* overexpression line) were germinated and grown for 4 days on 1/2 MS. The seedlings were transferred to 1/2 MS containing 0 mM NaCl (CK) and 100 mM NaCl (Salt), respectively. The morphological parameters were measured and the gene expression was analyzed after being grown vertically for 7 days. Total RNAs were extracted from the roots of seedlings of these genotypes and gene expression was determined by RT-qPCR using *AtActin2* mRNA as internal reference. Three independent repeats were done. The expression of *CdWRKY2* in Col-0 and *mapk3* was not detectable (ND). **(C)** LR number of different plant samples grown under control and salt conditions. Three independent experiments containing at least 10 plants were performed. Two-way ANOVA test was used and the data were further compared by Tukey’s post‐hoc test and statistically significant differences at *p* < 0.05 were displayed by different letters. **(D)** Image of different genotypes of plants samples grown under control and salt conditions. Bar = 1 cm.

Several reports have shown that WRKY TFs may function downstream of MAPK cascades. Rice OsWRKY30, which is the direct homologue of CdWRKY2, could interact with AtMAPK3 and was proved to play a vital role in drought tolerance of rice (Shen et al., 2012). Here, using the yeast two-hybrid method, we observed that CdWRKY2 could interact with AtMAPK3 in the yeast cell (**Figure 7A**). The expression level of CdWRKY2 was not detectable in Col-0 and *mapk3* (**Figure 7B**). Overexpression of *CdWRKY2* in the *mapk3* background *Arabidopsis* could partly rescue the salt oversensitive phenotype of LR grown on salt condition (**Figures 7C, D**) by statistical analysis of LR number between *CdWRKY2* overexpression lines in wild type and *mapk3* mutant background (**Figure 7C**). In the CdWRKY2 protein sequence, there are six potential SP (serine proline) phosphorylation sites (**Figure S8**). To identify the necessity of SP sites for the function of *CdWRKY2*, we mutated the first two SP sites (SP site into alanine proline site) and constructed an m*CdWRKY2* overexpression line in *Arabidopsis*. Results showed that the salt sensitivity of *mCdWRKY2* overexpression lines had no significant difference with the wild type (**Figures 7C, D**). These preliminary results suggested that the function of elevated salt sensitivity of *CdWRKY2* might partially be dependent on the function of MAPK3. Also, the SP sites of CdWRKY2 might play a critical role in the process of CdWRKY2 performing its function in elevating the salt sensitivity in *Arabidopsis*.

## 4 Discussion

WRKY has been reported to play multiple roles in plant development and abiotic responses including salt stress (Ülker and Somssich, 2004; Rushton et al., 2012). However, little progress in bermudagrass has been made because of its complex transformation and lack of genomic reference. In this study, a salt-induced *CdWRKY2* was cloned based on a reference sequence obtained by stitching two RNA-Seq data of bermudagrass (Hu et al., 2015; Shao et al., 2021). To investigate its function in salt response, we successfully obtained eight overexpression lines by transforming bermudagrass cultivar “Wrangler” with *pUbi::CdWRKY2* and two transgenic lines (CdOE13 and CdOE12) were further used for further study. Results showed that the overexpression lines exhibited enhanced salt sensitivity compared with the negative control lines (**Figure 2**), suggesting that CdWRKY2 might act as a negative regulator of salt tolerance in bermudagrass (Vanderauwera et al., 2012). We noticed that the root growth of control lines was significantly induced by salt compared to the control condition (**Figure 3**). This can be attributed to the relative salt-tolerant cultivar used in this study. However, under salt treatment, bermudagrass overexpressing *CdWRKY2* had a lower root growth rate than the negative control counterparts (**Figure 3**). We also noticed that the inhibition degree of overexpressing *CdWRKY2* on the primary root was relatively lower than that of the LR. To investigate the possible mechanism underlying the salt-induced LR inhibition of *CdWRKY2* overexpression lines, we then conducted overexpressed *Arabidopsis* lines of *CdWRKY2* (**Figures 4A, B**). The statistical results showed that both the AtOE10-4 and AtOE6-1 lines had less LR number (**Figure 4E**) than the wild type under salt treatment. However, the PR length was not obviously altered. Taken together, we speculated that the salt-induced inhibitory role of CdWRKY2 might be mainly exerted on the development of LR growth, which was reported to be a main determinant of plant root architecture (Péret et al., 2009).

Previous studies have shown that auxin metabolism plays an important role in LR development (Lavenus et al., 2013). To determine the possible effect of auxin distribution in salt-induced inhibitory on LR growth, we investigated the expression pattern of auxin-indicator gene *DR5::GUS* (Ulmasov et al., 1997) in the wild type (Col-0) and *CdWRKY2*-overexpressing (AtOE10-4) background *Arabidopsis* grown under both control and salt treatment. Compared with the wild type, the AtOE10-4 line displayed a weaker GUS staining in LR (**Figure 5**) especially under salt stress. To explore the possible mechanism, the expression of genes that participated in the major auxin synthesis pathway named IPA pathway was detected (Won et al., 2011; Zhao, 2012). We observed that the expression of auxin synthesis gene *YUC1* was highly expressed in *CdWRKY2*-overexpressing lines under both control and salt conditions and significantly decreased under salt conditions (**Figure 5D**). The expression of other auxin synthesis genes *TAA1*, *TAR2*, and *YUC4* had no obvious difference between the wild type and *CdWRKY2*-overexpressing lines under both control and salt conditions (**Figure 5E**; **S5E, F**). We also observed that *PIN3* was upregulated in *CdWRKY2*-overexpressing lines under both control and salt conditions. *PIN3* is expressed in the pericycle where LR is generated to function in auxin transportation (Friml et al., 2002). Thus, in this study, the upregulation of *PIN3* in *CdWRKY2-*overexpressing lines might also be a stimulus response during LR growth inhibition in *CdWRKY2*-overexpressing lines. In this study, CdWRKY2 could directly bind to the promoter of *AtWRKY46*, which was required for LR development under osmotic/salt stress by directly regulating the expression of auxin conjugation (to produce inactive forms of auxin to accurately modulate their endogenous auxin levels)-related gene *UGT84B2* and *GH3* in *Arabidopsis* (Staswick et al., 2005; Westfall et al., 2010; Korasick et al., 2013; Ding et al., 2015). The expression of *UGT84B2* was higher in *CdWRKY2*-overexpressing lines under both control and salt conditions (**Figure 5G**). However, *GH3.1* expression remained unchanged between the wild type and *CdWRKY2*-overexpressing lines under both control and salt conditions (**Figure S5H**), which might be probably because CdWRKY2 not only regulated the expression of *AtWRKY46* but also affected other modules to further neutralize the gene expression change of *GH3.1*.

ABA has previously been shown to play important roles in plants’ response to abiotic stress. Also, a series of TFs could function in abiotic response in an ABA-dependent manner including WRKY TFs (Zhou et al., 2008; Jiang and Deyholos, 2009; Cai et al., 2017). ABA has also been shown to play critical roles in mediating osmotic stress-dependent LR inhibition (Deak and Malamy, 2005). Here, we found that ABA significantly induced *CdWRKY2* expression in the roots of bermudagrass (**Figure S3B**) and the roots of *CdWRKY2-*overexpressing bermudagrass showed higher ABA sensitivity compared to their transgenic negative control (**Figures S3A, C, D**). To deduce whether CdWRKY2 elevated salt sensitivity of plants through the ABA pathway, we investigated the expression pattern of some well-characterized ABA-responsive genes in the roots of transgenic *Arabidopsis* lines (Lopez-Molina et al., 2001). We observed that *CdWRKY2* overexpression lines exhibited a remarkable altered expression of ABA-responsive genes (**Figures 6C–H**), suggesting an ABA-dependent function (Merlot et al., 2001; Reeves et al., 2011; Rushton et al., 2012). Reports indicate that ABI4 functions downstream of ABA signaling and plays important roles in ABA and cytokinin-mediated inhibition of LR formation by limiting *PIN1* expression, and thus reduces the polar auxin transport (Finkelstein et al., 1998; Signora et al., 2001; Shkolnik-Inbar and Bar-Zvi, 2010). A previous study also showed that AtWRKY46 could directly regulate the expression of *ABI4* in *Arabidopsis* to participate in LR development especially under osmotic/salt stress (Ding et al., 2015). Here, under both control and salt conditions, *ABI4* was highly expressed in transgenic lines compared to the wild type under both control and salt conditions (**Figure 6C**). In contrast to the salt condition, the expression of *PIN1* in the transgenic line was higher under normal conditions (**Figure 5F**). These results demonstrated that the *ABI4*-mediated limitation of auxin transport in the roots might be another route for LR growth inhibition and increased salt sensitivity. However, the *ABI4* expression was not significantly induced by salt, which might partly be due to the different sampling time used in this study. Together with the previous studies, here, CdWRKY2 and AtWRKY46 lacked protein interaction (**Figure S7**), but still CdWRKY2 might inhibit the expression level of *AtWRKY46*, which could further regulate several target genes downstream such as *ABI4* and *UGT84B2* to inhibit polar auxin transport and decrease root endogenous auxin levels especially under salt stress and lead to the inhibited LR growth (Ding et al., 2015).

MAPK cascade plays a critical role in plant stress response including salt stress (Cobb and Goldsmith, 2000). Several reports have shown that WRKY TFs may function downstream of MAPK cascades to participate in various physiological responses in plants (Asai et al., 2002; Kim and Zhang, 2004; Andreasson et al., 2005). In this study, we observed that CdWRKY2 could interact with AtMAPK3 in the yeast (**Figure 7A**). Furthermore, although the mutation of the *MAPK3* gene did not significantly inhibit the growth of LR in *Arabidopsis* under salt stress, overexpression of *CdWRKY2* in the *mapk3* background could partly rescue the salt oversensitive phenotype of LR grown on salt conditions (**Figures 7C, D**). A previous study showed that rice *OsWRKY30*, the orthologous gene of *CdWRKY2* (**Figure S9**) can be phosphorylated by OsMAPK3 to play vital roles in drought tolerance (Shen et al., 2012). The substrates of MAPK often contain several consecutive SP (serine proline) sites (Menke et al., 2005; Shen et al., 2012) and the mutation at SP site of the MAPK substrate could result in the loss of function WRKY in rice (Shen et al., 2012). In this study, we mutated the first two SP sites and constructed m*CdWRKY2* overexpression lines in *Arabidopsis*. The root salt sensitivity of *mCdWRKY2* overexpression lines remained statistically indifferent relative to the wild type. Also, there was no significant increase in LR number in the *mapk3* mutant compared to wild type under control and salt conditions (**Figures 7C, D**). These results at least suggested that the elevated LR salt sensitivity *via CdWRKY2* might partially be dependent on the function of MAPK3, and these SP sites might be the potential phosphorylation sites of MAPK3. Thus, whether the interaction between CdMAPK3 and CdWRKY2 exists in bermudagrass or not, as well as the phosphorylation of CdWRKY2, remains to be further examined. Moreover, whether OsWRKY30 has the same function as CdWRKY2 and whether knocking out *OsWRKY30* in rice could elevate the salt tolerance ability of mutants should be further investigated in rice. Moreover, phylogenetic analysis revealed that CdWRKY2 was the ortholog of AtWRKY2 in *Arabidopsis* (**Figures 1B**; **S1**). A previous study reported that the expression of *AtWRKY2* could be induced by NaCl and mannitol treatments, suggesting that AtWRKY2 might be involved in osmotic stress (Jiang and Yu, 2010). However, in the yeast one-hybrid assay, results showed that there was no interaction between the AtWRKY2 protein and the promoter of AtWRKY46 (**Figure 6J**), suggesting that AtWRKY2 and CdWRKY2 might not be direct counterparts during salt-induced root inhibition. However, based on the salt-response function of CdWRKY2, whether AtWRKY2 could participate in salt stress response in *Arabidopsis via* other pathways and the molecular mechanism in-depth should also be studied in the future.

In summary, we identified and overexpressed a salt-induced *WRKY* gene *CdWRKY2* in bermudagrass, which plays a negative role in salt-induced LR growth under salt stress. In *Arabidopsis*, CdWRKY2 could negatively regulate LR growth *via* the regulation of auxin homeostasis and ABA signaling-related genes while partly relying on the function of AtMAPK3 (**Figure S10**). Taken together, our results indicated that *CdWRKY2* and its homologue genes in other plant species should be useful targets for genetic engineering of salinity-tolerant plants to better utilize saline-alkali land.

## Data availability statement

The original contributions presented in the study are included in the article/**Supplementary Material**. Further inquiries can be directed to the corresponding author.

## Author contributions

AS and XX performed most of the experiments and wrote the article with contributions of all the authors. EA modified the manuscript. WW and SF provided technical assistance. YY, XL, GW, and HW provided experiment assistance. JF supervised the experiments. All authors contributed to the article and approved the submitted version.
