# Supplementary material for: CdWRKY2 transcription factor modulates salt oversensitivity in bermudagrass [Cynodon dactylon (L.) Pers.]
Source: Front Plant Sci. 2023 Jul 17;14:1164534. doi: 10.3389/fpls.2023.1164534 (PMC10388543; doi:10.3389/fpls.2023.1164534)
Supplement: Supplementary file 1 [file DataSheet_1.docx]

Supplementary Material

**CdWRKY2 transcription factor modulates salt over-sensitivity in bermudagrass (*Cynodon dactylon* (L.) Pers.)**

**An Shao^*^, Xiao Xu^*^, Amombo Erick, Wei Wang, Shugao Fan, Yanling Yin, Xiaoning Li, Guangyang Wang, Hongli Wang, Jinmin Fu^**^**

****Correspondence:** Jinmin Fu: [turfcn@qq.com](mailto:turfcn@qq.com))


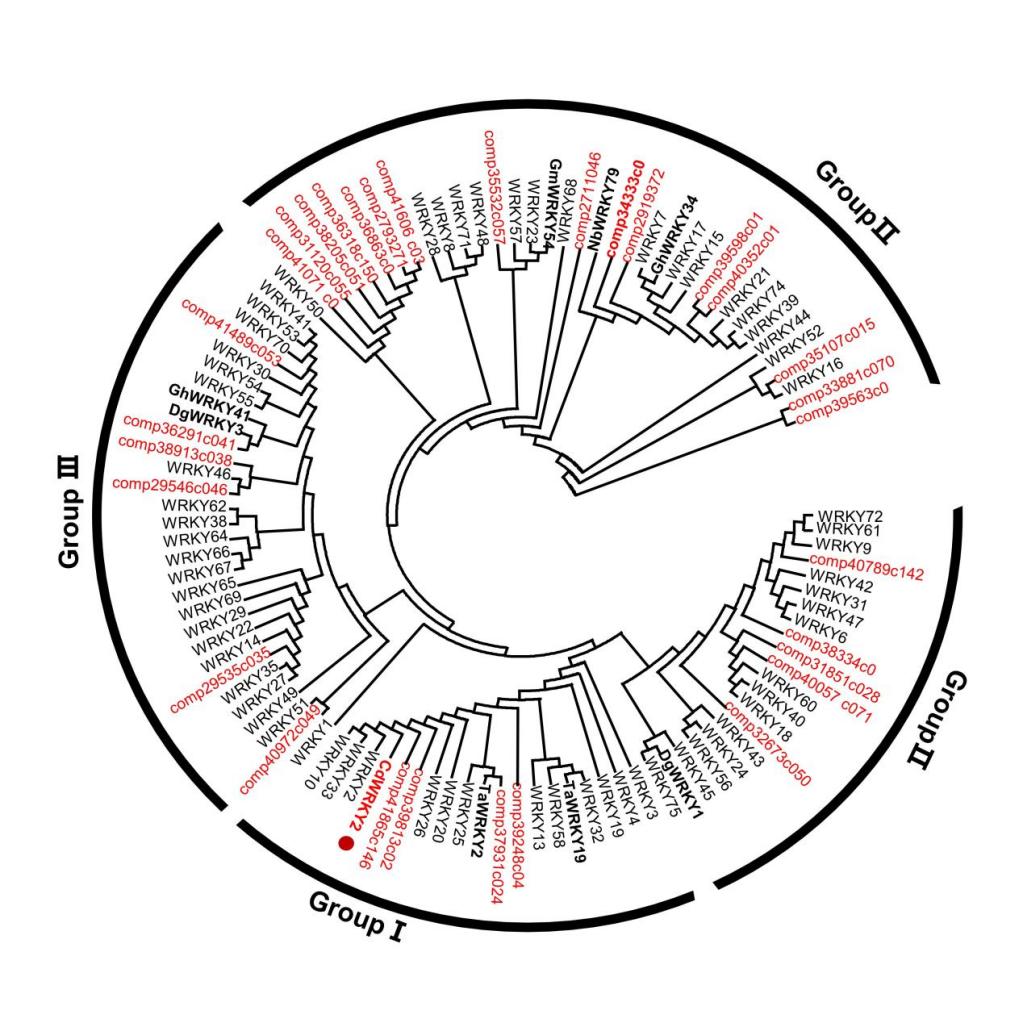


**Supplementary Figure 1. The phylogenetic analysis and subfamily clusters of some WRKY proteins in plants.** The sequences used for phylogenetic analysis include CdWRKY2 protein in bermudagrass and the some WRKY proteins in Soybean (GmWRKY54), Cotton (GhWRKY41, GhWRKY34), Wheat (TaWRKY2, TaWRKY19), Chrysanthemum (DgWRKY1, DgWRKY3), Nicotiana benthamiana (NbWRKY79) which were reported to function in salt response and all *Arabidopsis* WRKY proteins. CdWRKY2 from bermudagrass was shown in red color and boldface. The phylogenetic tree was constructed using the ClustalX program and the neighbor-joining method.


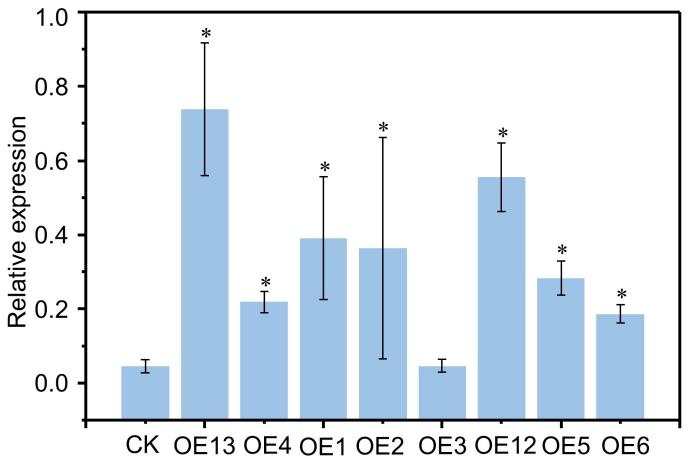


**Supplementary Figure 2. The CdWRKY2 relative expression levels of eight transgenic positive bermudagrass lines.** *CdActin2* was used as an internal control. Each value is the mean ± *SE* of three independent measurements. Significant differences between overexpression lines and transgenic control line are indicated by asterisk by Student’s *t*-test.

**Supplementary Figure 3. The phenotype of the *CdWRKY2* overexpression Bermudagrass lines under salt, salt+IAA, and ABA conditions.** A single branch of the wide type and overexpression lines were planted in soil for about 7 days.The seedlings were transferred to 1/2 Hogland containing 200 mM NaCl (Salt) , 200 mM NaCl with10 nM IAA (Salt+IAA) or 100 μM ABA for relative increase of root growth parameters measurement. (A) Images of the overexpression lines and control line grown under different conditions. Bar = 10 cm. (B) RT-qPCR analysis of *CdWRKY2* expression in the roots of bermudagrass in response to ABA (0, 3, 6, 12, 24 h). One-way ANOVA test was used and the data were further compared by Turkey’s post‐hoc test. Significant differences at *P*<0.05 were shown by different letters above the columns. (C) Relative increase of total roots length before and after treatment. (D) Relative increase of total roots number before and after treatment. Data are the means ± *SD* of four biological replicates. Two-way ANOVA test was used and the data were further compared by Turkey’s post‐hoc test. Significant differences at *P* < 0.05 were shown by different letters above the columns.

**Supplementary Figure 4. Relative GUS activity of roots expressing DR5::GUS in Col-0 and AtOE10-4 under control and salt condition.** GUS activity of plants was normalized to that of the untreated Col-0 background plants. Two-way ANOVA test was used and the data were further compared by Turkey’s post‐hoc test. Different letters on histograms indicate that means were statistically different at the *P*< 0.05 level.

**Supplementary Figure 5. The relative expression of auxin biosynthesis related, auxin conjugation related, and transport related genes in *35S::CdWRKY2* overexpression *Arabidopsis* lines and wild type Col-0.** *UBQ10* was used as an internal reference. Three independent repeats were performed. Two-way ANOVA test was used and the data were further compared by Turkey’s post‐hoc multiple range test. Different letters on histograms indicate that means were statistically different at the *P*< 0.05 level.

**Supplementary Figure 6. The *ABI1* and *ABI2* expression in *35S::CdWRKY2* overexpression *Arabidopsis* lines.** Relative expression of *ABI1* (A) and *ABI2* (B) in Col-0 and overexpression lines under control and salt conditions. *UBQ10* was used as an internal reference. Data are means ± *SD* three independent experiments. Two-way ANOVA test was used and the data were further compared by Turkey’s post‐hoc multiple range test. Different letters on histograms indicate that means were statistically different at the *P*< 0.05 level.


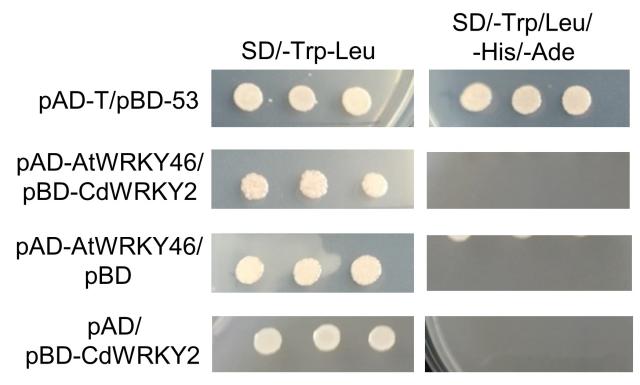


**Supplementary Figure 7. No interactions between AtWRKY46 and CdWRKY2 in the yeast.** Transformants transferred with different construct pairs growth on SD/-Trp-Leu and SD/-Trp-Leu-His-Ade plates. pGADT7-T (pAD-T)/pGBKT7-53 (pBD-53) was used as positive control vector pair.


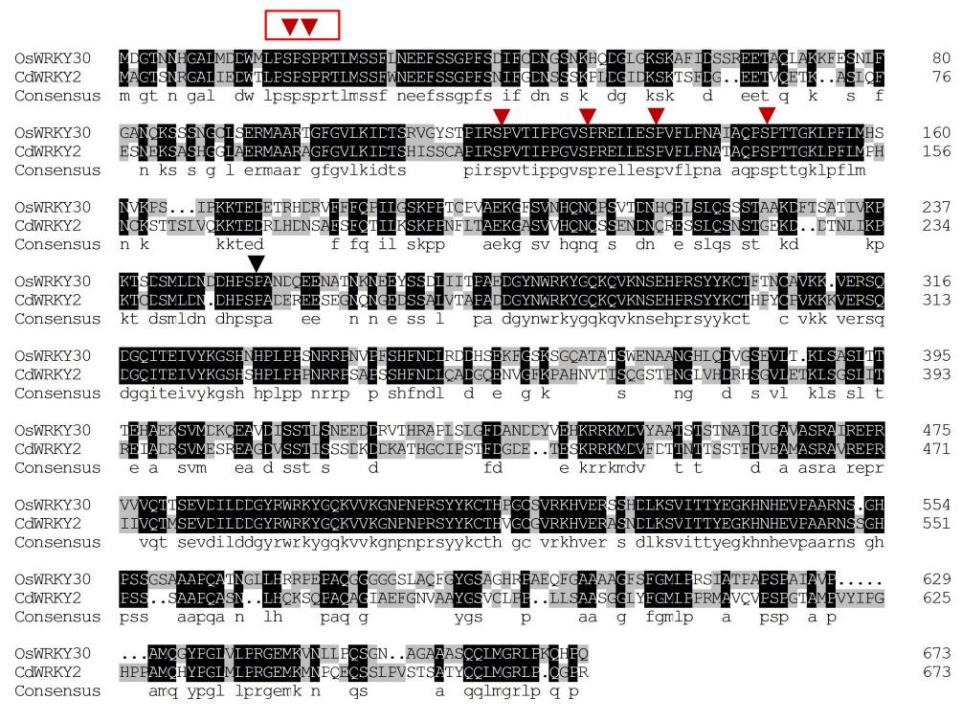


**Supplementary Figure 8. The mutated SP sites displayed on CdWRKY2 protein sequence.** Red triangles indicate six SP sites in the N-terminal of CdWRKY2 and two SP sites in the red box were mutated. The mutant was introduced from the primers used for amplification and serine proline site SP was mutated into alanine proline site to further generate *35S::mCdWRKY* overexpression lines.


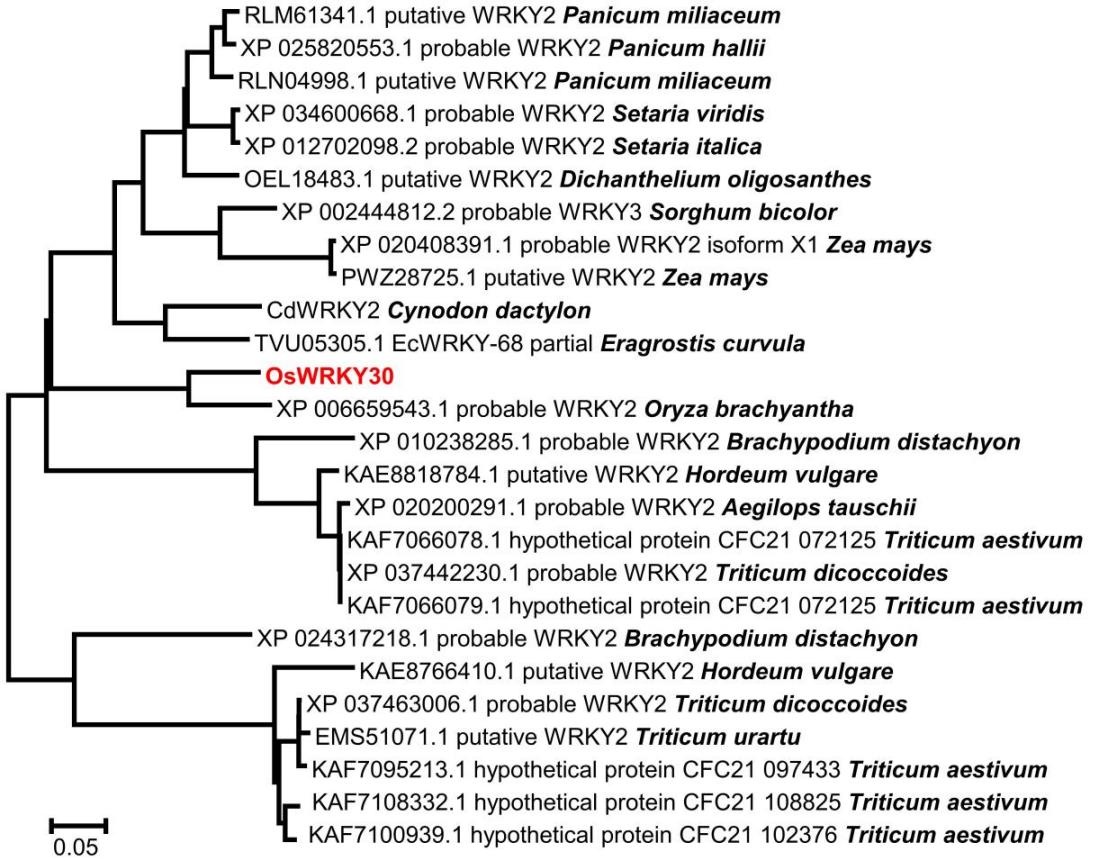


**Supplementary Figure 9. The phylogenetic of some OsWRKY30 homologue proteins in plants.** The sequences used for phylogenetic analysis include OsWRKY30 in rice, CdWRKY2 protein in bermudagrass and the some WRKY proteins in *Panicum miliaceum*, *Setaria viridis*, *Dichanthelium oligosanthes*, *Sorghum bicolor*, *Zea mays*, *Eragrostis curvula*, *Oryza brachyantha*, *Brachypodium distachyon*, *Hordeum vulgare*, *Aegilops tauschii*, *Triticum aestivum*, *Triticum dicoccoides*, *Triticum urartu*. OsWRKY30 from rice was shown in red color and boldface. The phylogenetic tree was constructed using the Clustal X program and the neighbor-joining method.


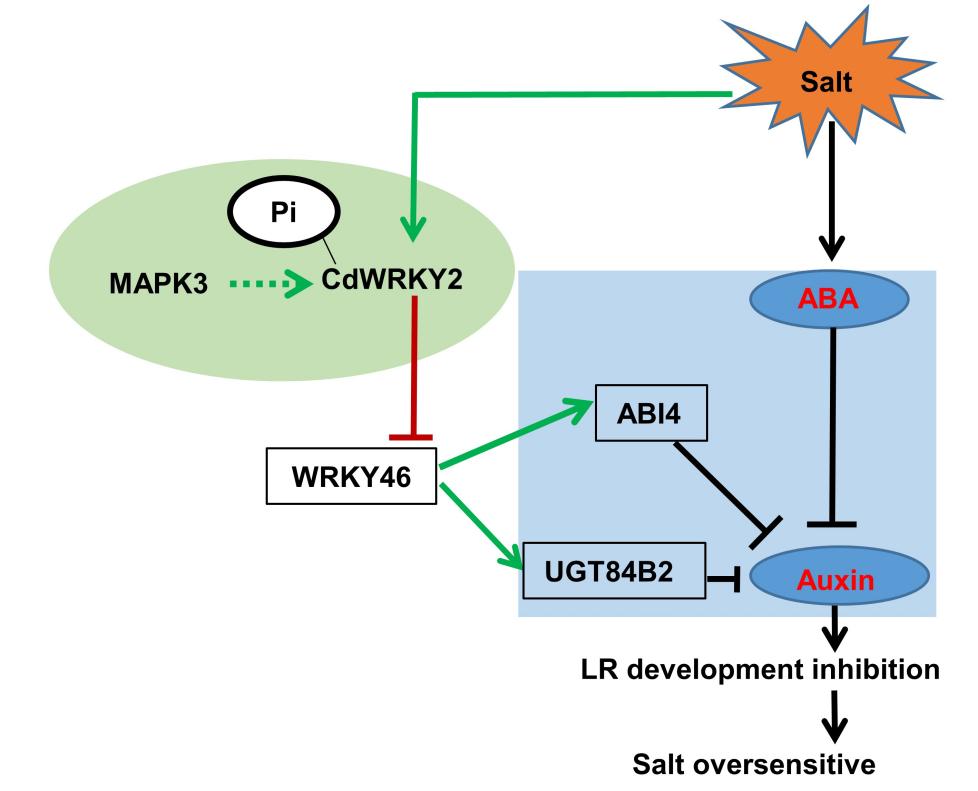


**Supplementary Figure 10. A proposed model of CdWRKY2 transformed into *Arabidopsis* participated in salt-induced lateral root growth inhibition.** CdWRKY2 negatively regulate the expression level of *AtWRKY46* and then might further regulate several target genes downstream such as *ABI4* and *UGT84B2* to inhibit polar auxin transport and decrease root endogenous auxin levels and lead to the inhibited LR growth under salt stress. CdWRKY2 could negatively regulate LR growth under salt stress via regulation of ABA signaling and auxin homeostasis and partly rely on the function of AtMAPK3.

**Supplementary Table 1. The primers used for vectors construction.**

| **Primer name** | **Primer sequence (5′-3′)** | | **Vectors** |
| --- | --- | --- | --- |
| UCdWRKY2 | AGCAGGCTTTGACTTTATGGCGGGAACTAGCAACCGTGGAG | GCTGGGTCTAGAGACTT  TTAAATCCGAGGACCCTGAGGCAACCTG | *PGWC-CdWRKY2* |
| 35CdWRKY2 | CCCGGGATGGCGGGAACTAGCAACCGTGGAG | GGATCCTTAAATCCGAGGACCCTGAGGCAACCTG | *35S::CdWRKY2* |
| ADCdWRKY2 | GTACCAGATTACGCTCATATGATGGCGGGAACTAGCAACCGTGGAG | CAGCTCGAGCTCGATGGATCCTTAAATCCGAGGACCCTGAGGCAACCTG | *PGADT7::CdWRKY2* |
| BDCdWRKY2 | TCAGAGGAGGACCTGCATATGATGGCGGGAACTAGCAACCGTGGAG | CCGCTGCAGGTCGACGGATCCTTAAATCCGAGGACCCTGAGGCAACCTG | *PGBKT7::CdWRKY2* |
| ADMAPK3 | GTACCAGATTACGCTCATATGATGGATGCCAAGCGCACGCTC | CAGCTCGAGCTCGATGGATCCCTGCTCAAATTCGAACGAGAA | *PGADT7::MAPK3* |
| ADWRKY46 | GTACCAGATTACGCTCATATGATGATGATGGAAGAGAAACT TGTGA | CAGCTCGAGCTCGATGGATCCCTACGACCACAACCAATCCTGTCCG | *PGADT7::WRKY46* |
| mCdWRKY2 | CCCGGGATGGCGGGAACTAGCAACCGTGGAGCTCTGATAGAAGATTGGACGCTTCCCGCACCCGCC | GGATCCTTAAATCCGAGGACCCTGAGGCAACCTG | *35S::mCdWRKY2* |
| *pABI4* | GGTACCGGGTAATTCATGCCACGCTTT | GTCGACGAGGAAGTAGAGAGAGAAAG | *pABI4*::*AbAi* |
| *pUGT84B2* | GGTACCACCTCATGCTTCATTACCTATTT | GTCGACTCTTGACCCTCATTACTTCCCAT | *pUGT84B2*::*AbAi* |
| *pWRKY46* | GGTACCATCCATAGATTCTTGATTTGCTT | GTCGACGTCCTTGTGCTTAGACAATAAGT | *pWRKY46*::*AbAi* |

**Supplementary Table 2. The primers used for quantitative real-time PCR.**

| **primer name** | **primer sequence (5′-3′)** | |
| --- | --- | --- |
| *q-CdWRKY46* | CGTCGGAATACATCGTCAGC | AGTTGGTCGGAGGAACATCA |
| *q-* *CdActin2* | TCTGAAGGGTAAGTAGAGTAG | ACTCAGCACATTCCAGCAGAT |
| *q-* *CdPP2A* | ACTACTTCCCCTTGACGGCA | AAATACCCAACCACATCGA |
| *q-CdWRKY2* | GCAGCTTCAGGTGGTCTC | CGGATGTCCAGGAATGTA |
| *q-AtWRKY46* | ACATCACATCCCCGAAGACG | ACTTCTTCGGACTTGGTCGG |
| *q-AtActin2* | CCTTCGTCTTGATCTTGCGG | AGCGATGGCTGGAACAGAAC |
| *q-AtUBQ10* | GATCTTTGCCGGAAAACAA | GAAGATCTGCATACCCACGC |
| *q-TAA1* | GATGAAGAATCGGTGGGAGAAGC | CGTCCCTAGCCACGCAAACGCAGG |
| *q-TAR2* | CATGATTTGGCTTACTATTGGCCA | GTCTTTCACCAAAGCCCATCCA |
| *q-YUC1* | ATGGAGTCTCATCCTCACAACAAA | AAGGGACTCCACGGCTCGAG |
| *q-YUC4* | ATGGGCACTTGTAGAGAATCAGA | TAACCGATGGTACGCCTCGGTTT |
| *q-PIN1* | CCTCAGGGGAATAGTAACGACA | TCATCGTCTTTGTTACCGAAAC |
| *q-PIN2* | GGCGAAGAAAGCAGGAAGA | GGTGGGTACGACGGAACA |
| *q-PIN3* | CCCAGATCAATCTCACAACG | CCGGCGAAACTAAATTGTTG |
| *q-PIN7* | TGGGCTCTTGTTGCTTTCA | TCACCCAAACTGAACATTGC |
| *q-PID* | ATATGGCAAGACTCCGTTCG | GTTAAGCAACCCGGAAATCA |
| *q-UGT84B2* | GAAAGGCGAAAACGTCCAGG | ATCCAAGTCGGATACGCCAC |
| *q-GH3.1* | AGGCAGCCAAGTCATGAGAC | TGCACCTCTTGAGATTGCGT |
| *q-IAGLU* | AGCCCAAGGTCACATCAACC | GGGTTTCGGGGACGTTTTCT |
| *q-ABI3* | GGCAGGGATGGAAACCAGAAAAG | GGCAAAACGATCCTTCCGAGGT |
| *q-ABI4* | TCAATAACTCATCCACCGCCGTTG | AGGCCAAATGGTCGAAGATCCA |
| *q-ABI5* | ATTGGCGGAGTTGGAGAGGAAGA | TCGGTTGTGCCCTTGACTTCAA |
| *q-ABF1* | CGAAAACAGGCTTATACCTTGG | GCCTGTTTCTTCTGCAAATCTT |
| *q-ABF2* | TCATCAGAAGGGATAGGGAAGA | CCTCTCACACCACCATTAAACA |
| *q-ABF3* | GTGTGTCTCGCTTCTTCCCTAT | TCAACCTGCATGAAACATTTG |
| *q-ABI1* | AGAGTGTGCCTTTGTATGGTTT | CATCCTCTCTCTACAATAGTTCG |
| *q-ABI2* | GATGGAAGATTCTGTCTCAACGA | CTTTCTCCTTCACTATCTCCTCC |

**Supplementary Table 3. The individual *P* value of physiological parameters in Figure 2 and Figure 3 by two way ANOVA , Tukeys post-hoc test.**

| Index  P value  Genotype  Pairs | | Withering rate | Biomass | Plant height | EL | POD | CAT | SOD | MDA | PR length  Increase | Total root length  Increase | Total root number  Increase |
| --- | --- | --- | --- | --- | --- | --- | --- | --- | --- | --- | --- | --- |
| OE12  /CK | OE12/Salt | 0.993 | 0.001 | 0 | 0.003 | 0.001 | 0.007 | 0.914 | 0.848 | 0.008 | 0.237 | 0.988 |
|  | OE13/CK | 0.019 | 0.572 | 0.587 | 0.858 | 0.774 | 0.992 | 0.983 | 0.993 | 0.708 | 0.996 | 1 |
|  | OE13/Salt | 0.007 | 0.002 | 0 | 0 | 0 | 0.01 | 1 | 0.192 | 0.005 | 0.999 | 1 |
|  | WT/CK | 0.011 | 0.001 | 0.985 | 0.766 | 0.001 | 0.018 | 0.838 | 0.978 | 0.998 | 0.759 | 1 |
|  | WT/Salt | 0.004 | 0.374 | 0.005 | 0.39 | 0 | 0.023 | 0.414 | 0.511 | 0 | 0 | 0 |
| OE12  /Salt | OE12/CK | 0.993 | 0.001 | 0 | 0.003 | 0.001 | 0.007 | 0.914 | 0.848 | 0.008 | 0.237 | 0.988 |
|  | OE13/CK | 0.008 | 0 | 0 | 0 | 0 | 0.017 | 0.587 | 0.559 | 0.088 | 0.449 | 0.954 |
|  | OE13/Salt | 0.003 | 1 | 0.692 | 0.691 | 0.92 | 1 | 0.803 | 0.746 | 1 | 0.134 | 0.96 |
|  | WT/CK | 0.004 | 0 | 0 | 0 | 1 | 0.99 | 0.32 | 0.997 | 0.004 | 0.895 | 0.995 |
|  | WT/Salt | 0.002 | 0 | 0.002 | 0.081 | 0.262 | 0.972 | 0.916 | 0.987 | 0.099 | 0 | 0 |
| OE13  /CK | OE12/CK | 0.019 | 0.572 | 0.587 | 0.858 | 0.774 | 0.992 | 0.983 | 0.993 | 0.708 | 0.996 | 1 |
|  | OE12/Salt | 0.008 | 0 | 0 | 0 | 0 | 0.017 | 0.587 | 0.559 | 0.088 | 0.449 | 0.954 |
|  | OE13/Salt | 0.988 | 0 | 0 | 0 | 0 | 0.027 | 0.998 | 0.082 | 0.055 | 0.949 | 1 |
|  | WT/CK | 0.999 | 0.023 | 0.271 | 1 | 0 | 0.047 | 0.994 | 0.81 | 0.473 | 0.951 | 0.999 |
|  | WT/Salt | 0.943 | 0.999 | 0.073 | 0.071 | 0 | 0.06 | 0.164 | 0.259 | 0.001 | 0 | 0 |
| OE13  /Salt | OE12/CK | 0.007 | 0.002 | 0 | 0 | 0 | 0.01 | 1 | 0.192 | 0.005 | 0.999 | 1 |
|  | OE12/Salt | 0.003 | 1 | 0.692 | 0.691 | 0.92 | 1 | 0.803 | 0.746 | 1 | 0.134 | 0.96 |
|  | OE13/CK | 0.988 | 0 | 0 | 0 | 0 | 0.027 | 0.998 | 0.082 | 0.055 | 0.949 | 1 |
|  | WT/CK | 1 | 0 | 0 | 0 | 0.966 | 0.999 | 0.936 | 0.49 | 0.003 | 0.548 | 1 |
|  | WT/Salt | 1 | 0 | 0.021 | 0.007 | 0.758 | 0.996 | 0.292 | 0.972 | 0.154 | 0 | 0 |
| WT  /CK | OE12/CK | 0.011 | 0.001 | 0.985 | 0.766 | 0.001 | 0.018 | 0.838 | 0.978 | 0.998 | 0.759 | 1 |
|  | OE12/Salt | 0.004 | 0 | 0 | 0 | 1 | 0.99 | 0.32 | 0.997 | 0.004 | 0.895 | 0.995 |
|  | OE13/CK | 0.999 | 0.023 | 0.271 | 1 | 0 | 0.047 | 0.994 | 0.81 | 0.473 | 0.951 | 0.999 |
|  | OE13/Salt | 1 | 0 | 0 | 0 | 0.966 | 0.999 | 0.936 | 0.49 | 0.003 | 0.548 | 1 |
|  | WT/Salt | 0.993 | 0.043 | 0.002 | 0.052 | 0.338 | 1 | 0.072 | 0.881 | 0 | 0 | 0 |
| WT  /Salt | OE12/CK | 0.004 | 0.374 | 0.005 | 0.39 | 0 | 0.023 | 0.414 | 0.511 | 0 | 0 | 0 |
|  | OE12/Salt | 0.002 | 0 | 0.002 | 0.081 | 0.262 | 0.972 | 0.916 | 0.987 | 0.099 | 0 | 0 |
|  | OE13/CK | 0.943 | 0.999 | 0.073 | 0.071 | 0 | 0.06 | 0.164 | 0.259 | 0.001 | 0 | 0 |
|  | OE13/Salt | 1 | 0 | 0.021 | 0.007 | 0.758 | 0.996 | 0.292 | 0.972 | 0.154 | 0 | 0 |
|  | WT/CK | 0.993 | 0.043 |  | 0.052 | 0.338 | 1 | 0.072 | 0.881 | 0 | 0 | 0 |

**Supplementary Table 4. The individual *P* value of gene expression comparison in Figure 4 and Figure 5 by two way ANOVA , Tukeys post-hoc test.**

| Gene  P Value  Genotype  Pairs | | *YUC1* | *YUC4* | *PIN1* | *UGT84B2* | *ABI4* | *ABI5* | *ABI3* | *ABF1* | *ABF2* | *ABF3* |
| --- | --- | --- | --- | --- | --- | --- | --- | --- | --- | --- | --- |
| Col/CK | Col/Salt | 1 | 1 | 0.001 | 0.998 | 0.889 | 0 | 0.904 | 0 | 0.06 | 0.552 |
|  | OE10-4/CK | 0 | 0.069 | 0.002 | 0.502 | 0 | 0.933 | 0.002 | 0.729 | 0.882 | 0.196 |
|  | OE10-4/Salt | 0.014 | 0.145 | 0.965 | 0 | 0.679 | 0.003 | 0.913 | 1 | 0.08 | 1 |
|  | OE6-1/CK | 0 | 0.034 | 0.003 | 0.042 | 0 | 0.958 | 0.003 | 0.675 | 0.872 | 0.155 |
|  | OE6-1/Salt | 0.004 | 0.063 | 0.968 | 0 | 0.616 | 0.001 | 0.888 | 1 | 0.057 | 0.999 |
| Col/Salt | Col/CK | 1 | 1 | 0.001 | 0.998 | 0.889 | 0 | 0.904 | 0 | 0.06 | 0.552 |
|  | OE10-4/CK | 0 | 0.105 | 1 | 0.302 | 0 | 0 | 0 | 0 | 0.01 | 0.012 |
|  | OE10-4/Salt | 0.018 | 0.214 | 0 | 0 | 0.187 | 0 | 0.394 | 0 | 1 | 0.397 |
|  | OE6-1/CK | 0 | 0.052 | 0.986 | 0.021 | 0 | 0 | 0.001 | 0 | 0.009 | 0.009 |
|  | OE6-1/Salt | 0.006 | 0.095 | 0 | 0 | 0.158 | 0 | 0.359 | 0 | 1 | 0.75 |
| OE10-4/CK | Col/CK | 0 | 0.069 | 0.002 | 0.502 | 0 | 0.933 | 0.002 | 0.729 | 0.882 | 0.196 |
|  | Col/Salt | 0 | 0.105 | 1 | 0.302 | 0 | 0 | 0 | 0 | 0.01 | 0.012 |
|  | OE10-4/Salt | 0.004 | 0.997 | 0 | 0.007 | 0 | 0.015 | 0.011 | 0.857 | 0.013 | 0.297 |
|  | OE6-1/CK | 0.987 | 0.998 | 0.999 | 0.577 | 0.989 | 1 | 1 | 1 | 1 | 1 |
|  | OE6-1/Salt | 0.011 | 1 | 0 | 0.007 | 0 | 0.006 | 0.012 | 0.809 | 0.009 | 0.114 |
| OE10-4/Salt | Col/CK | 0.014 | 0.145 | 0.965 | 0 | 0.679 | 0.003 | 0.913 | 1 | 0.08 | 1 |
|  | Col/Salt | 0.018 | 0.214 | 0 | 0 | 0.187 | 0 | 0.394 | 0 | 1 | 0.397 |
|  | OE10-4/CK | 0.004 | 0.997 | 0 | 0.007 | 0 | 0.015 | 0.011 | 0.857 | 0.013 | 0.297 |
|  | OE6-1/CK | 0.01 | 0.944 | 0.001 | 0.115 | 0 | 0.013 | 0.015 | 0.814 | 0.013 | 0.24 |
|  | OE6-1/Salt | 0.98 | 0.994 | 1 | 1 | 1 | 0.994 | 1 | 1 | 1 | 0.985 |
| OE6-1/CK | Col/CK | 0 | 0.034 | 0.003 | 0.042 | 0 | 0.958 | 0.003 | 0.675 | 0.872 | 0.155 |
|  | Col/Salt | 0 | 0.052 | 0.986 | 0.021 | 0 | 0 | 0.001 | 0 | 0.009 | 0.009 |
|  | OE10-4/CK | 0.987 | 0.998 | 0.999 | 0.577 | 0.989 | 1 | 1 | 1 | 1 | 1 |
|  | OE10-4/Salt | 0.01 | 0.944 | 0.001 | 0.115 | 0 | 0.013 | 0.015 | 0.814 | 0.013 | 0.24 |
|  | OE6-1/Salt | 0.032 | 0.999 | 0.001 | 0.115 | 0 | 0.005 | 0.017 | 0.76 | 0.009 | 0.089 |
| OE6-1/Salt | Col/CK | 0.004 | 0.063 | 0.968 | 0 | 0.616 | 0.001 | 0.888 | 1 | 0.057 | 0.999 |
|  | Col/Salt | 0.006 | 0.095 | 0 | 0 | 0.158 | 0 | 0.359 | 0 | 1 | 0.75 |
|  | OE10-4/CK | 0.011 | 1 | 0 | 0.007 | 0 | 0.006 | 0.012 | 0.809 | 0.009 | 0.114 |
|  | OE10-4/Salt | 0.98 | 0.994 | 1 | 1 | 1 | 0.994 | 1 | 1 | 1 | 0.985 |
|  | OE6-1/CK | 0.032 | 0.999 | 0.001 | 0.115 | 0 | 0.005 | 0.017 | 0.76 | 0.009 | 0.089 |

**Supplementary Table 5. The individual *P* value of gene expression multiple comparison in Figure S3 and Figure S4 by two way ANOVA , Tukeys post-hoc test.**

| Gene  P Value  Genotype  Pairs | | *PIN3* | *PIN2* | *PIN7* | *PID* | *TAA1* | *TAR2* | *IAGLU* | *GH3.1* | *ABI1* | *ABI2* |
| --- | --- | --- | --- | --- | --- | --- | --- | --- | --- | --- | --- |
| Col/CK | Col/Salt | 0.002 | 0.919 | 1 | 0.093 | 0.013 | 0 | 0.874 | 0.95 | 0.019 | 0.069 |
|  | OE10-4/CK | 0.862 | 0.946 | 1 | 0.54 | 0.705 | 0.031 | 1 | 0.216 | 0.099 | 0.157 |
|  | OE10-4/Salt | 0.002 | 0.999 | 1 | 0.061 | 0.018 | 0 | 0.995 | 0.493 | 0 | 0 |
|  | OE6-1/CK | 0.621 | 0.906 | 0.611 | 0.62 | 0.7 | 0.169 | 1 | 0.166 | 0.702 | 0.352 |
|  | OE6-1/Salt | 0.001 | 1 | 1 | 0.178 | 0.003 | 0 | 0.991 | 0.374 | 0 | 0 |
| Col/Salt | Col/CK | 0.002 | 0.919 | 1 | 0.093 | 0.013 | 0 | 0.874 | 0.95 | 0.019 | 0.069 |
|  | OE10-4/CK | 0.014 | 1 | 1 | 0.802 | 0.001 | 0.003 | 0.955 | 0.622 | 0 | 0.995 |
|  | OE10-4/Salt | 1 | 0.781 | 1 | 1 | 1 | 1 | 0.616 | 0.924 | 0.027 | 0 |
|  | OE6-1/CK | 0.03 | 1 | 0.642 | 0.729 | 0.001 | 0.001 | 0.962 | 0.522 | 0.002 | 0.883 |
|  | OE6-1/Salt | 1 | 0.931 | 1 | 0.998 | 0.928 | 1 | 0.57 | 0.836 | 0.046 | 0 |
| OE10-4/CK | Col/CK | 0.862 | 0.946 | 1 | 0.54 | 0.705 | 0.031 | 1 | 0.216 | 0.099 | 0.157 |
|  | Col/Salt | 0.014 | 1 | 1 | 0.802 | 0.001 | 0.003 | 0.955 | 0.622 | 0 | 0.995 |
|  | OE10-4/Salt | 0.009 | 0.828 | 1 | 0.664 | 0.002 | 0.002 | 0.97 | 0.986 | 0 | 0 |
|  | OE6-1/CK | 0.997 | 1 | 0.577 | 1 | 1 | 0.894 | 1 | 1 | 0.669 | 0.992 |
|  | OE6-1/Salt | 0.009 | 0.955 | 1 | 0.953 | 0 | 0.002 | 0.954 | 0.998 | 0 | 0 |
| OE10-4/Salt | Col/CK | 0.002 | 0.999 | 1 | 0.061 | 0.018 | 0 | 0.995 | 0.493 | 0 | 0 |
|  | Col/Salt | 1 | 0.781 | 1 | 1 | 1 | 1 | 0.616 | 0.924 | 0.027 | 0 |
|  | OE10-4/CK | 0.009 | 0.828 | 1 | 0.664 | 0.002 | 0.002 | 0.97 | 0.986 | 0 | 0 |
|  | OE6-1/CK | 0.021 | 0.761 | 0.542 | 0.583 | 0.002 | 0 | 0.963 | 0.961 | 0 | 0 |
|  | OE6-1/Salt | 1 | 0.999 | 1 | 0.982 | 0.855 | 1 | 1 | 1 | 0.999 | 0.646 |
| OE6-1/CK | Col/CK | 0.621 | 0.906 | 0.611 | 0.62 | 0.7 | 0.169 | 1 | 0.166 | 0.702 | 0.352 |
|  | Col/Salt | 0.03 | 1 | 0.642 | 0.729 | 0.001 | 0.001 | 0.962 | 0.522 | 0.002 | 0.883 |
|  | OE10-4/CK | 0.997 | 1 | 0.577 | 1 | 1 | 0.894 | 1 | 1 | 0.669 | 0.992 |
|  | OE10-4/Salt | 0.021 | 0.761 | 0.542 | 0.583 | 0.002 | 0 | 0.963 | 0.961 | 0 | 0 |
|  | OE6-1/Salt | 0.019 | 0.919 | 0.546 | 0.915 | 0 | 0 | 0.945 | 0.991 | 0 | 0 |
| OE6-1/Salt | Col/CK | 0.001 | 1 | 1 | 0.178 | 0.003 | 0 | 0.991 | 0.374 | 0 | 0 |
|  | Col/Salt | 1 | 0.931 | 1 | 0.998 | 0.928 | 1 | 0.57 | 0.836 | 0.046 | 0 |
|  | OE10-4/CK | 0.009 | 0.955 | 1 | 0.953 | 0 | 0.002 | 0.954 | 0.998 | 0 | 0 |
|  | OE10-4/Salt | 1 | 0.999 | 1 | 0.982 | 0.855 | 1 | 1 | 1 | 0.999 | 0.646 |
|  | OE6-1/CK | 0.019 | 0.919 | 0.546 | 0.915 | 0 | 0 | 0.945 | 0.991 | 0 | 0 |

**The CDS sequence of *CdWRKY2***

ATGGCGGGAACTAGCAACCGTGGAGCTCTGATAGAAGATTGGACGCTTCCCTCACCCAGCCCAAGAACACTAATGTCAAGCTTCTGGAATGAAGAATTCAGCTCTGGTCCATTCTCCAACATTTTCGGCGACAACAGTAGTAGCAAGCCCCTGGATGGAATTGATAAGAGCAAAACTTCCTTTGATGGGGAAGAAACTGTGCAAGAAACAAAAGCCTCCCTCCAGTTTGAATCCAATGAGAAATCAGCCTCACACGGCGGTCTTGCCGAAAGGATGGCTGCAAGGGCTGGTTTTGGCGTTCTGAAAATTGATACATCCCATATCAGTTCATGTGCACCAATTCGATCACCTGTGACCATTCCCCCTGGTGTGAGCCCACGAGAACTTCTTGAGTCGCCTGTTTTTCTTCCCAATGCCACTGCGCAACCTTCTCCTACCACTGGTAAACTGCCATTTCTGATGCCTCACAACTGTAAATCAACGACATCATTAGTCCAAAAGAAGACTGAAGATCGCTTACATGACAATTCTGCATTTTCCTTCCAGACGATATTGAAGTCTAAACCACCAAACTTTCTGACTGCAGAAAAGGGTGCAAGTGTTGTTCACCAAAACCAGTCCTCAGAAAATGATAATCAGCGGGAGTCAAGTCTTCAATCTAACTCTACTGGGGAAAAGGATGACACAAACCTTATCAAACCTAAGACGTGTGATTCAATGTTGGACAATGATCATCCTTCCCCTGCCGATGAACGAGAAGAAAGTGAGGGAAACCAAAATGGGGAGGACTCTTCAGCTCTAGTCACAGCTCCTGCTGACGATGGATATAACTGGAGAAAATACGGACAAAAACAAGTTAAGAACAGTGAGCATCCAAGAAGCTACTATAAATGTACTCATCCATATTGTCCTGTCAAGAAAAAGGTCGAACGTTCTCAAGATGGTCAAATAACAGAGATAGTGTACAAAGGTTCTCATAGTCACCCTTTGCCGCCTCCCAACCGCCGGCCAAGTGCCCCTTCGTCGCACTTCAATGACTTGCAAGCTGATGGCCAGGAGAATGTTGGTTTCAAACCTGCCCATAACGTAACAATTTCACAGGGAAGCACCCCAAATGGCCTTGTCCACGATAGGCACAGTGGAGTTCTTGAAACAAAGCTGTCTGGTTCTCTCATCACACGAGAGATTGCTGACAGATCTGTTATGGAGTCTCGAGAAGCTGGAGATGTTTCCTCAACAATCTCCTCTAGTGACAAGGATGACAAGGCAACACACGGTTGTATTCCTTCGACCTTCGATGGGGATGAGACTGAGTCAAAAAGAAGGAAGATGGATGTTTTTGACACAACCAACACTACCAGCAGCACCTTTGATGTGGAAGCTATGGCATCAAGGGCTGTCAGGGAGCCTCGGATTATTGTGCAAACCATGAGTGAGGTCGACATCCTTGATGATGGTTACCGCTGGCGCAAGTATGGGCAAAAAGTTGTCAAAGGAAATCCAAACCCAAGGAGCTACTACAAATGCACGCATGTGGGATGCGGGGTGCGCAAGCATGTGGAGAGAGCTTCAAATGATCTCAAATCTGTCATCACGACATATGAGGGCAAGCACAACCATGAAGTTCCAGCTGCTAGAAATAGTAGCGGGCATCCAAGCTCCAGCGCTGCACCACAGGCAAGCAATCTTCACCAGAAGTCACAACCGGCTCAAGCCGGCATTGCAGAGTTCGGCAATGTTGCTGCCTATGGTTCAGTTTGTCTCCCACCACTACTCAGTGCAGCTTCAGGTGGTCTCTACTTCGGAATGCTCCCGCCTCGCATGGCAGTTCAGGTACCATCTCCTGGAACCGCCATGCCTGTGTACATTCCTGGACATCCGCCAGCAATGCAGCATTACCCAGGGCTTATGCTGCCAAGAGGTGAGATGAAGATGAACCCACAGGAGCAGTCCAGCTTGCCAGTATCAACCTCGGCAACATACCAGCAGCTCATGGGCAGGTTGCCTCAGGGTCCTCGGATTTAA
